# Identification and Profiling of MicroRNAs from Skeletal Muscle of the Common Carp

**DOI:** 10.1371/journal.pone.0030925

**Published:** 2012-01-27

**Authors:** Xuechun Yan, Lei Ding, Yunchao Li, Xiaofeng Zhang, Yang Liang, Xiaowen Sun, Chun-Bo Teng

**Affiliations:** 1 Key Laboratory of Aquatic Biotechnology, Heilongjiang River Fisheries Research Institute, Chinese Academy of Fishery Sciences, Harbin, China; 2 College of Life Sciences, Northeast Forestry University, Harbin, China; Auburn University, United States of America

## Abstract

The common carp is one of the most important cultivated species in the world of freshwater aquaculture. The cultivation of this species is particularly productive due to its high skeletal muscle mass; however, the molecular mechanisms of skeletal muscle development in the common carp remain unknown. It has been shown that a class of non-coding ∼22 nucleotide RNAs called microRNAs (miRNAs) play important roles in vertebrate development. They regulate gene expression through sequence-specific interactions with the 3′ untranslated regions (UTRs) of target mRNAs and thereby cause translational repression or mRNA destabilization. Intriguingly, the role of miRNAs in the skeletal muscle development of the common carp remains unknown. In this study, a small-RNA cDNA library was constructed from the skeletal muscle of the common carp, and Solexa sequencing technology was used to perform high throughput sequencing of the library. Subsequent bioinformatics analysis identified 188 conserved miRNAs and 7 novel miRNAs in the carp skeletal muscle. The miRNA expression profiling showed that, miR-1, miR-133a-3p, and miR-206 were specifically expressed in muscle-containing organs, and that miR-1, miR-21, miR-26a, miR-27a, miR-133a-3p, miR-206, miR-214 and miR-222 were differentially expressed in the process of skeletal muscle development of the common carp. This study provides a first identification and profiling of miRNAs related to the muscle biology of the common carp. Their identification could provide clues leading towards a better understanding of the molecular mechanisms of carp skeletal muscle development.

## Introduction

The common carp (*Cyprinus Carpio L.*) occupies a prominent position in the world of freshwater aquaculture and serves as a major source of animal protein for millions of people [1]. In spite of its aquacultural importance and a production increase in carp breeding over the last decade, the current productive species are mainly selected through traditional breeding methods, including the use of mutual hybrids and backcrosses to domesticated ancestors [2]. Molecular techniques are still not widely used in the breeding of the common carp due to a lack of genetic and genomic information. In recent years, several carp traits, including cold tolerance, body weight and plasma glucose contents, have been studied through development of molecular markers and construction of genetic linkage maps [3]–[5]. Although it is an important economic trait, the skeletal muscle mass of the common carp has not been well defined by molecular markers or control mechanisms to date.

Several lines of evidence in mouse and zebrafish models have suggested that the development of vertebrate skeletal muscle is orchestrated by evolutionarily conserved gene expression networks in which transcription factors play important roles in regulating muscle growth and differentiation [6]. An essential myogenic transcription factor is the MADS box transcription factor myocyte enhancer factor-2 (MEF2), which activates the myogenic differentiation program in conjunction with the basic-helix-loop-helix (bHLH) transcription factors MyoD and myogenin in skeletal muscle [7]. Recent studies have shown that, in addition to transcriptional factors involved in muscle proliferation and differentiation, a set of microRNAs (miRNAs) also play important roles in skeletal muscle development in vertebrate animals [8], [9].

miRNAs are a class of non-coding RNAs of approximately 22 nucleotides in length, which are processed from stem-loop RNA precursors (pre-miRNAs, ∼65 nucleotides) by the ribonuclease III (RNase III) enzyme Dicer [10]. The pre-miRNAs are excised from the longer primary transcripts (pri-miRNAs) in the nucleus by the RNAse III enzyme Drosha [11]. The mature miRNAs exert their functions by incorporating one of the duplex strands into the RNA-induced silencing complexes (RISCs) and then binding the 3′ untranslated region (3′UTR) of the target mRNAs to degrade or repress their translation [12]. Many studies have demonstrated that miRNAs exist in a wide range of invertebrates and vertebrates, and that they are involved in the regulation of animal development, physiological functions and pathological processes [13]–[16].

miRNAs can be identified through manual cloning and sequencing, miRNA array screening, and bioinformatic approaches [17], [18]. Recently, high throughput sequencing technology has been widely used to facilitate the identification and detection of miRNAs in multiple species [19]–[23]. For this study, we constructed a small-RNA cDNA library from the skeletal muscle of the common carp. Through high throughput sequencing of the small RNA library and subsequent bioinformatic analysis, miRNAs in skeletal muscle tissue of the common carp were identified. Finally, using expression profiling analysis of the miRNAs from different tissues and multiple development stages, miRNAs likely to play a role in carp muscle development were identified.

## Results

### Construction of cDNA library for sequencing and small RNA discovery

**Figure 1:**
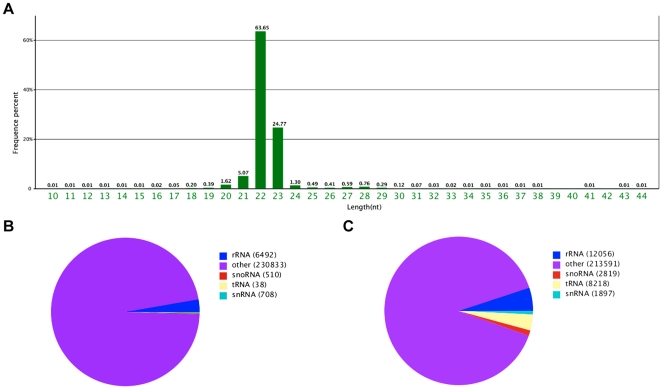
Anaylsis of small RNAs derived from the skeletal muscle of the common carp. A: Length distribution of sequenced small RNAs; B: The clean reads were blasted against the GenBank database to annotate rRNA, RNA, snRNA, or snoRNA; C: The clean reads were blasted against the Rfam database noncoding RNA to annotate rRNA, RNA, snRNA, or snoRNA.

The cDNA library of small RNAs was constructed with pooled total RNAs from skeletal muscle tissues collected from three different 1-year-old common carps. Through high throughput Solexa sequencing, 21335605 total reads were obtained; 93.49% of the reads were 21–23 nucleotides in length (Figure 1A). After removal of the 5′ and 3′ adapters, pollution reads and reads smaller than 18 nucleotides, 20842912 high-quality clean reads were extracted, and 25966 of the resulting unique small RNAs were annotated as either rRNA, snRNA, snoRNA, or tRNA using BLAST searches against the NCBI Genebank and Rfam database (Figure 1B, 1C) and then removed from the following analysis. The remainder of the unannotated small RNAs was mapped to the zebrafish genome, which is the only completely sequenced genome of the *Cyprinidae* fish family, to perform distribution analysis using SOAP (http://soap.genomics.org.cn). 35815 unique sequences (16965286 reads) were perfectly mapped to the zebrafish genome.

### Identification of miRNAs in the skeletal muscle of common carp

To identify conserved miRNAs in the skeletal muscle tissue of the common carp, the small RNAs with a length of 18–23 nucleotides were searched against the latest miRBase release version 17.0 (miRBase V17.0). 2422 unique sequences (16522982 reads) were annotated as miRNA candidates, while the rest (3916545 reads) were unannotated. The 2422 miRNA candidates were then clustered into 188 categories according to sequence similarity. Each category included multiple homologs, which differed in sequence length by only 1–5 nucleotides. Such homologous sequences with different lengths are thought to be variants produced by various biochemical modifications and by imprecise processing of primary or precursor miRNAs by Drosha and Dicer enzymes. The sequence with the highest read in a category was considered to be the representative sequence for the following analysis.

**Figure 2:**
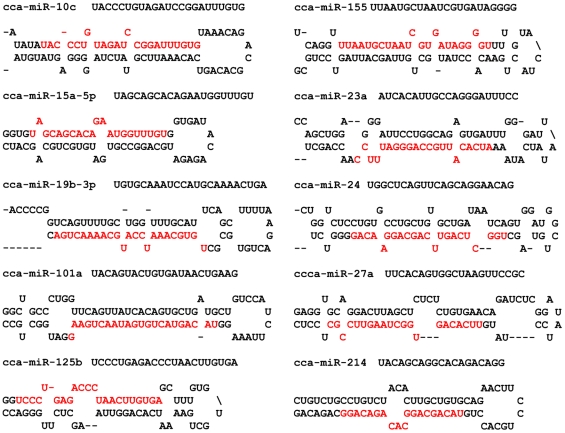
Prediction of the fold-back structure of 10 carp miRNA precursors. The precursor sequences were obtained by sequence alignment with the sequences of the common carp genome and ESTs. The mature miRNA sequences in the precursors are indicated in red.

Apart from the characteristic length (∼22 nucleotides) and conservation, an important criteria for annotating a cloned sequence as a miRNA is the presence of a compact pre-miRNA fold-back structure [24]. Since we are sequencing the genome of the common carp ourselves and aligning the sequence to the chromosomes, we selected 20 candidate miRNA sequences to compare to the sequenced ESTs and genome sequence of the common carp and predict possible precursor sequences. Subsequently, we predicted the miRNA precursor structure using mfold software and found that all the 20 sequences could form miRNA precursors with stem-loop structure (Figure 2, Figure S1), indicating that annotating the cloned sequences as miRNAs through conservation analysis is quite reliable. The 188 conserved sequences were annotated as common carp miRNAs (Table S1).

The unannotated small RNAs that could be mapped to the zebrafish genome were subjected to novel miRNA prediction analysis of their secondary structure, the Dicer enzyme cleavage site and the minimum free energy using Mireap software (Beijing Genomics Institute). 15 specific reads of ∼22 nucleotides in length were considered miRNA candidates. These sequences were further searched against the ESTs and genome sequence of the common carp in an attempt to predict possible precursor sequences. 7 miRNA precursors with stem-loop structure were found using mfold software (Figure S2); thus 7 novel and specific miRNAs were identified from the skeletal muscle tissue of the common carp (Table 1).

**Table 1:** Novel miRNAs identified in the common carp.

miRNA	Sequence	Frequency
CM1-m0001_3p	UGGAAGUGAAUGGUGACUGAG	12
CM1-m0002_3p	GCGACCCAUCCUUGGUUUCUG	12
CM1-m0003_5p	ACCAUGGCUGUAGACUGUUACC	42
CM1-m0004_3p	UCAAGGUCCGCUGUGAACACGA	8
CM1-m0005_3p	UGAGGAGUUUAGAGCAAGUAA	107
CM1-m0006_3p	UAGUUUGAUUCACAGCACAAGA	17
CM1-m0007_3p	UCAAGGUCCGCCGUGAACACGC	12

### Conservation analysis of the identified common carp miRNAs

To date, although the miRBase V17.0 has included miRNAs from 84 different animal species, miRNA members from non-model animals have not been fully identified. To analyze the conservation of common carp miRNAs, we compared them to those identified in model animals, including worm (*Caenorhabditis elegans*), fly (*Drosophila melanogaster*), frog (*Xenopus tropocalis*), zebrafish (*Danio rerio*), chicken (*Gallus gallus*), mouse (*Mus musculus*) and human (*Homo sapiens*). Only let-7, miR-1, and miR-139 are highly conserved from worm to human, but 96 common carp miRNAs are conserved in all vertebrate model animals (Table S1), although there exist base edition and length change of sequences across the different animal species. Moreover, 31 common carp miRNAs are only identified in teleosts (Table 2). The comparison was performed between the miRNAs discovered in the common carp with those identified in the bighead carp and silver carp [23], and the result shows that 80% common carp miRNAs are discovered in the bighead carp and silver carp (Table S3).

**Table 2:** Common carp miRNAs only conserved in the teleosts.

miRNA	Sequence	Frequency	Conserved
cca-let-7h	UGAGGUAGUAAGUUGUGUUGU	159	dre fru tni hno hmo
cca-miR-10d	UACCCUGUAGAACCGAAUGUGU	10	dre fru tni ola hno hmo
cca-miR-27e	UUCACAGUGGCUAAGUUCAGU	126	dre fru tni hno hmo
cca-miR-153b	UUGCAUAGUCACAAAAAUGAGC	6	dre fru tni hno hmo
cca-miR-460-3p	CACAGCGCAUACAAUGUGGAUG	7	dre fru tni ola
cca-miR-142b-5p	CAUAAAGUAGACAGCACUACU	8	dre fru tni hno hmo
cca-miR-430	UAAGUGCUAUUUGUUGGGGUAG	2	dre ola hno hmo
cca-miR-125c	UCCCUGAGACCCUAACUCGUGA	7625	dre ola hno hmo
cca-miR-18c	UAAGGUGCAUCUUGUGUAGUUAG	64	dre hno hmo
cca-miR-19d	UGUGCAAACCCAUGCAAAACUGA	59	dre ola hno hmo
cca-miR-27d	UUCACAGUGGCUAAGUUCUUC	22	dre ola hno hmo
cca-miR-454b	UAGUGCAAUAUUGCUUAUAGG	36	dre hno hmo
cca-miR-462	UAACGGAACCCAUAAUGCAGCUG	1518	dre ola hno hmo
cca-miR-722	UUUUUUGCAGAAACGUUUCAG	53	dre hno hmo
cca-miR-724	UUAAAGGGAAUUUGCGACUGUU	65	dre hno hmo
cca-miR-725	UUCAGUCAUUGUUUCUAGUAGU	58	dre hno hmo
cca-miR-726	UUCACUACUAGCAGAACUCGG	19	dre
cca-miR-730	UCCUCAUUGUGCAUGCUGUGUG	154	dre
cca-miR-731	AAUGACACGUUUUCUCCCGGAUC	97	dre ola hno hmo
cca-miR-135c	UAUGGCUUUCUAUUCCUAUGUGA	7	dre hno hmo
cca-miR-153c	UUGCAUAGUCACAAAAAUGAUC	2	dre hno hmo
cca-miR-734	UAAAUGCUGCAGAAUCGUACCG	7	dre hno hmo
cca-miR-18b	UAAGGUGCAUUUAGUGCAGAUAG	3	dre
cca-miR-457a	AGCAGCACAUCAAUAUUGGC	4	dre hno hmo
cca-miR-457b	AGCAGCACAUAAAUACUGGAG	1	dre hno hmo
cca-miR-459-5p	AGUAACAAGGAUUCAUCCUGUU	4	dre ola
cca-miR-727-3p	GUUGAGGCGAGUUGAAGACUUA	4	dre hno hmo
cca-miR-729	CAUGGGUAUGAUACGACCUGGG	1	dre
cca-miR-738	GCUACGGCCCGCGUCGGGA	1	dre
cca-miR-27c-5p	CAGGACUUAACCCACUUGUGAAC	310	dre
cca-miR-459-3p	CAGGGAAUCUCUGUUACUGGG	4	dre ola

dre: *Danio rerio*; ola: *Oryzias latipes*; fru: *Fugu rubripes*; tni: *Tetraodon nigroviridis*; hno: *Hypophthalmichthys nobilis*; hmo: *Hypophthalmichthys molitrix*.

**Figure 3:**
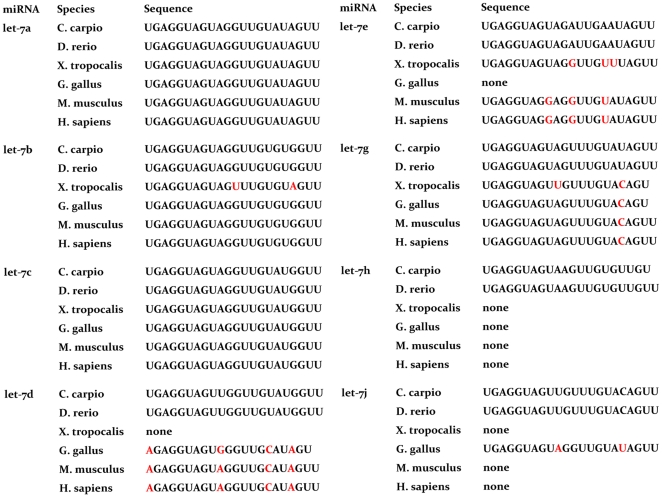
Comparation of the member sequences of the let-7 family in model vertebrate animals. Bases that differ between model vertebrate animals are indicated in red.

Let-7, one of the most conserved miRNAs, has evolved into a miRNA family that includes 8 members in the common carp. Of these, 5 members possess base or length edition between different vertebrate model animals but no changes in the seed sequence (Figure 3). Apart from let-7, 32 other miRNA families were found to be included in the 188 conserved common carp miRNAs. Of these, only cca-miR-16c, cca-miR-193b, cca-miR-200c, and cca-miR-301c have differences in the seed sequence from their respective family members (Table S2).

Through literature mining, we found that 27 miRNAs are known to be involved in animal muscle development [8], [25]–[47], among them 16 miRNAs were found in the common carp (Table 3). Conservation analysis showed that the 16 miRNAs were highly conserved in multiple animal species (Table S1).

**Table 3:** The miRNAs both identified in the common carp and demonstrated to be muscle-related in several species.

miRNA	Sequence	Frequency	Species
miR-1	UGGAAUGUAAAGAAGUAUGUAU	1850549	mmu [29] ,hsa [32] ,dre [33]
miR-125b	UCCCUGAGACCCUAACUUGUGA	6170	mmu [34]
miR-133a-3p	UUGGUCCCCUUCAACCAGCUGU	1506	mmu [29] dre [33]
miR-155	UUAAUGCUAAUCGUGAUAGGGG	109	mmu [26]
miR-181a-5p	AACAUUCAACGCUGUCGGUGA	14161	mmu [35]
miR-206	UGGAAUGUAAGGAAGUGUGUGG	7555941	mmu [31], [36], [37] hsa [32], [38]
miR-21	UAGCUUAUCAGACUGGUGUUGGC	2082419	rno [25]
miR-214	UACAGCAGGCACAGACAGG	267	mmu [39], [40] dre [41]
miR-221	AGCUACAUUGUCUGCUGGG	26	mmu [42]
miR-222	AGCUACAUCUGGCUACUGGG	45	mmu [42]
miR-23a	AUCACAUUGCCAGGGAUUUCC	130	hsa [43]
miR-24	UGGCUCAGUUCAGCAGGAACAG	3027	mmu [44]
miR-26a	UUCAAGUAAUCCAGGAUAGGCU	8278	mmu [45]
miR-27a	UUCACAGUGGCUAAGUUCCGC	558	mmu [46]
miR-27b	UUCACAGUGGCUAAGUUCUGC	2814	mmu [46], [47]
miR-29b	UAGCACCAUUUGAAAUCAGUGUU	129	mmu hsa [48]

dre, *Danio rerio*; mmu, *Mus musculus*; hsa, *Homo sapiens*.

### Validation and expression analysis of the identified common carp miRNAs

**Figure 4:**
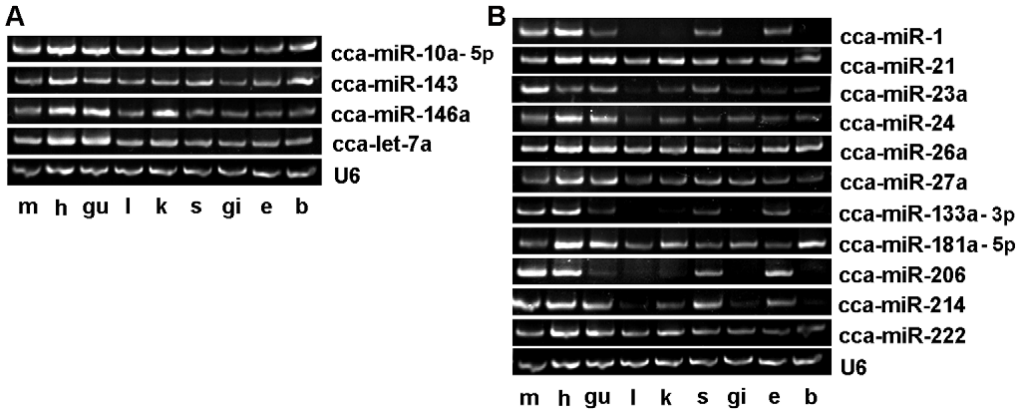
The expression of miRNAs in tissues and organs of the common carp were detected by RT-PCR. A: Several high-read miRNAs were detected in skeletal muscle (m), heart (h), gut (gu), liver (l), kidney (k), skin (s), gill (gi), eye (e), and brain (b) of the common carp; B: Eleven muscle-related miRNAs were detected in skeletal muscle (m), heart (h), gut (gu), liver (l), kidney (k), skin (s), gill (gi), eye (e), and brain (b) of the common carp. Expression of miRNAs was normalized to U6.

For validation and identification of muscle-related miRNAs in the common carp, RT-PCR analysis of miRNA expression was performed in skeletal muscle, heart, gut, liver, kidney, skin, gill, eye, and brain of the common carp. We first randomly picked out several high-read miRNAs, including let-7a, miR-10a-5p, miR-143, and miR-146a. The results showed that all 4 miRNAs could be amplified from the skeletal muscle, heart, gut, liver, kidney, skin, gill, eye, and brain, and that the expression levels of these miRNAs were relatively consistent across all 9 tissue types (Fig. 4A). These results indicate that let-7a, miR-10a-5p, miR-143, and miR-146a are ubiquitously expressed in multiple tissues and organs.

We then selected miR-1, miR-21, miR-23a, miR-24, miR-26a, miR-27a, miR-133a-3p, miR-181a-5p, miR-206, miR-214, and miR-222, which have been reported to play roles in the development of vertebrate skeletal muscle (Table 3). Of these, miR-1, miR-133a-3p, and miR-206, were found to be specifically expressed in heart, skeletal muscle, gut, eye, and skin (Fig. 4B), whereas miR-21, miR-26a, miR-181a-5p, and miR-222 were detected in all the samples with little expression level change. Importantly, miR-23a, miR-24, miR-27a, and miR-214 were highly expressed in muscle-related tissue or organs (skeletal muscle, heart, gut), although they could also be detected in non-muscle organs (Fig. 4B). These muscle-specific and highly expressed miRNAs may therefore play roles in the skeletal muscle development of the common carp.

### Profiling of the identified common carp miRNAs at different developmental stages

**Figure 5:**
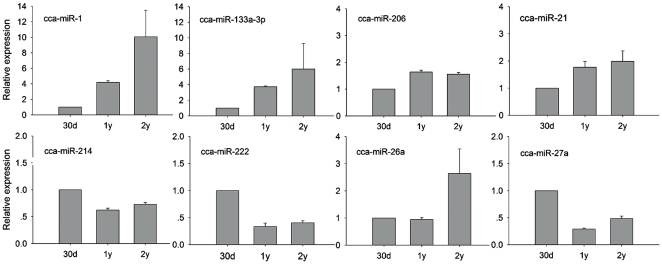
The expression of muscle-related miRNAs during skeletal muscle development of the common carp was detected by qRT-PCR. The profile of miRNAs was measured at the skeletal muscle tissues of 30 day post-hatching (dph) larva, 1-year old and 2-year old common carps. The level of miRNA expression in the skeletal muscles of 30 dph larva was defined as 1. 30 d, 30 dph skeletal muscles; 1 y, 1-year skeletal muscles; 2 y, 2-year skeletal muscles. At least 5 animals were used for each timepoint.

To further explore the miRNAs involved in the skeletal muscle development of the common carp, we performed quantitative analysis of the miRNAs in skeletal muscle tissues of 30 day post-hatching (dph) larva, 1-year-old common carps, and 2-year-old common carps. The results showed that the expression of miR-1, miR-133a-3p, miR-206, and miR-21 was increased in the skeletal muscles from 30 day post-hatching to 2-year-old (Figure 5). Among four miRNAs, miR-1 and miR-133a-3p were expressed at significantly higher levels in the 2-year skeletal muscles than 1-year skeletal muscles (Figure 5). MiR-26a expression was not changed in the 30 dph and 1-year skeletal muscles, but increased in the 2-year skeletal muscles (Figure 5). These results suggest that miR-1, miR-133a-3p, miR-21, miR-26, and miR-206 play roles in the growth and functional maintenance of carp skeletal muscles. miR-27a, miR-214, and miR-222 are expressed at relatively high levels in the 30 dph skeletal muscles, and then decreased in the 1-year and 2-year skeletal muscles (Figure 5). These results indicate that miR-27a, miR-214, and miR-222 are related to the process of skeletal muscle proliferation and differentiation in the common carp.

The expression of miRNAs was also analyzed at different developmental stages, including fertilized oocytes, 24 hour post-fertilization (hpf) embryos, 48 hpf embryos, 1 dph larva, 7 dph larva, 15 dph larva, and 30 dph larva. The results showed that miR-1, miR-133a-3p, and miR-206 were increased expression 48 hour post fertilization, and the other miRNAs were increased expression 7 day post hatching (Figure S3). Of note, half of detected miRNAs (miR-21, miR-26a, miR-27a, and miR-214) were expressed at a higher level in the fertilized oocytes than in embryos after fertilization (Figure S3); this is likely caused by the existence of maternal miRNAs or dicer.

## Discussion

Vertebrate skeletal muscle develops through the proliferation and terminal differentiation of somatic cells committed to myogenic lineage [48]. Multiple transcriptional factors and miRNAs are known to be involved in skeletal myogenesis in mouse and zebrafish models [8], [9]. However, the molecular mechanisms of skeletal muscle development in the common carp are not well understood. In this study, we focused on the identification and profiling of miRNAs involved in the development of carp skeletal muscle. Using Solexa sequencing technology and bioinformatic analysis, 188 conserved miRNAs and 7 novel miRNAs were identified from carp skeletal muscle. Among the 188 conserved miRNAs, 97 are conserved in all vertebrate model animals. Moreover, the closer is the evolutionary link between two species, the more conserved their miRNA sequences are. Of the 27 miRNAs that have previously been reported to be involved in muscle development, 16 were found in the common carp and multiple animal species. These highly conserved miRNAs might be expected to perform similar functions in carp muscle development as they do in model animal species.

Profiling of miRNAs expression in the common carp indicate that miR-1, miR-133a-3p, and miR-206 are specifically expressed in the muscle-rich tissues and organs (skeletal muscle, heart, gut) in the common carps, and that their expression increases with the progress of common carp muscle development, ultimately reaching their highest levels in the skeletal tissues of mature carp. These results are consistent with previous findings in mouse, zebrafish and pig [29], [30], [32], [49]. It has been reported that a loss of Dicer function or a down-regulation of miR-1 and miR-133 in zebrafish alters muscle gene expression and disrupts actin organization during sarcomere assembly [32]. And studies in vitro have shown that miR-1, miR-133, and miR-206 can target multiple muscle-development-related genes, including Fstl1, Utrn [50], HDAC4 [29], Pax7 [30], c-Met [31], [37], SRF [29]. Although the carp-specific target genes of miR-1, miR-133, and miR-206 are not known, their specific expression pattern and high conservation indicate that they are also likely to play a role in the development of carp skeletal muscle.

Other muscle-related miRNAs in vertebrates, including miR-21, miR-214, miR-26, miR-27, miR-221, and miR-222, have also been identified in the common carp. miR-21 is a key determinant in the IL-11/Stat3 anti-apoptotic signaling pathway involved in the preconditioning of skeletal myoblasts [25], miR-26a targets Ezh2 during myogenesis [44], miR-27 regulates behaviors of muscle stem cells by repression of Pax3 expression [45], miR-214 promotes myogenic differentiation by facilitating exit from mitosis via down-regulation of proto-oncogene N-ras [38], and miR-221 and miR-222 modulate differentiation and maturation of skeletal muscle cells by targeting p27 [41]. However, whether these miRNAs influence carp muscle development by targeting similar genes requires further investigation.

## Materials and Methods

### Animals and Tissue collection

All animal experiments were performed using wild common carps bred in the Heilongjiang Fishery Institute in accordance with the recommendations in the Guide for the Care and Use of Laboratory Animals of China. The study was approved by the Committee on the Ethics of Animal Experiments of the Heilongjiang Fishery Institute (Permit Number: 11-1970).

Male and female adult common carp were separately kept in tanks with oxygen-saturated water at 23–25°C. Pituitary extract was used to induce and synchronize ovulation and spermiation. Artificial propagation was performed in hatcheries, and the incubation of early embryos was carried out in hatchery jars. Hatched fry were kept in a large glass tanks at room temperature. The experimental materials include fertilized oocytes, 24 hour post-fertilization (hpf) embryos, 48 hpf embryos, 1 day post-hatching (dph) larva, 7 dph larva, 15 dph larva, 30 dph larva, skeletal muscle tissues from 30 dph larva, 1-year-old and 2-year-old common carp. Tissues including skeletal muscle, skin, heart, liver, kidney, gill, gut, eye, and brain were collected by dissection from 1-year and 2-year-old common carp. The samples were snap-frozen in liquid nitrogen and stored at −80°C.

### Small RNA isolation and cDNA library construction

Skeletal muscle tissues used for the generation of the small RNA library were obtained from 3 individual 1-year-old fish. Total RNA was isolated from skeletal muscle using TRIzol reagent (Invitrogen). Subsequently, the population of recovered small-RNAs, ranging in size from 18 to 30 nucleotides was purified from 15% polyacrylamide gel, and these small RNAs were ligated with the 3′ and 5′ adapter ligation. Reverse transcription reactions were performed using the RT primer, and PCR reactions were performed using the forward and reverse primers. The PCR product was purified via phenol/chloroform extraction and ethanol precipitation and was delivered to the Beijing Genomics Institute to be sequenced using Solexa technologies.

### Sequence analysis and identification of miRNAs

Initial reads obtained from Solexa sequencing were processed by removing poor quality reads, 5′ adapter pollution reads, reads without 3′ adapter, reads without insert fragment, reads containing poly(A) stretches, and reads less than 18 nt. The clean reads were blasted against the Rfam database (http://www.sanger.ac.uk/software/Rfam) and the GenBank noncoding RNA database (http://blast.ncbi.nlm.nih.gov/) to annotate rRNA, RNA, snRNA and snoRNA. The other small RNAs were mapped to the zebrafish genome to perform distribution analysis and miRNA prediction using SOAP (http://soap.genomics.org.cn).

miRNA identification was performed by comparing the sequenced small RNAs with the known miRNAs of zebrafish or other animal species in miRBase v17.0. The miRNA precursors with a stem-loop structure were predicted by homologous comparison miRNA sequences to ESTs and to the genome sequence of the common carp with mfold software (http://mfold.rna.albany.edu/?q=mfold/RNA-Folding-Form).

### RT-PCR and qRT-PCR

Total RNAs were isolated from embryonic and adult samples using TRIzol reagent (Invitrogen) and then treated with DNase I (RNase-free) (Takara). To detect miRNA expression, total RNAs were polyadenylated with ATP by E. coli poly (A) polymerase (Biolabs, New England). The polyadenylated total RNAs were reverse transcribed with M-MLV Reverse Transcription Reagents (Invitrogen) and a poly (T) primer ligated with a RACE adapter for miRNA quantitative assays. The RNAs without polyadenylation were reverse transcribed with oligo d (T) for the mRNA assay.

qRT-PCR was performed using the ABI 7500 sequence detection system (Applied Biosystems). U6 small nuclear RNA was used as an endogenous control for miRNAs. All reactions were run in triplicate and included no template controls for each gene. The relative amount of miRNA to U6 RNA was calculated using the 2^−ΔΔCT^ method, and the level of significance was determined by one-way analysis of variance (ANOVA) with SPSS Statistics 17.0. All quantitative data presented were the mean ± SEM. All primers for RT-PCR and qPCR were shown in supplementary materials (Table S4).

## Supporting Information

Figure S1 **Prediction of the fold-back structure of 10 conserved carp miRNA precursors.** The precursor sequences were obtained by sequence alignment with the sequences of the common carp genome and ESTs. The mature miRNA sequences in the precursors are indicated in red.
(TIF)

Figure S2 **Prediction of the precursor structure of 7 novel carp miRNAs.** The precursor sequences were obtained by sequence alignment with the sequences of the common carp genome and ESTs. The mature miRNA sequences in the precursors are indicated in red.
(TIF)

Figure S3 **The expression of muscle-related miRNAs during development of the common carp was detected by qRT-PCR.** 0 h, fertilized oocytes; 24 h, 24 hpf embryos; 48 h, 48 hpf embryos; 1 d, 1 dph larva; 7 d, 7 dph larva; 15 d, 15 dph larva; 30 d, 30 dph larva. At least 5 animals were used for each timepoint. The level of miRNA expression in the 30 dph larva was defined as 1.
(TIF)

Table S1 **Highly-conserved miRNAs identified in the common carp.**
(DOC)

Table S2 **miRNA families identified in the common carp.**
(DOC)

Table S3 **Comparison between the discovered miRNAs in the common carp and those found in the bighead carp and silver carp.**
(DOC)

Table S4 **Primer sequences for RT-PCR and qPCR assays.**
(DOC)
