# Supplementary figures and images for: Identification and Profiling of MicroRNAs from Skeletal Muscle of the Common Carp
Source: PLoS One. 2012 Jan 27;7(1):e30925. doi: 10.1371/journal.pone.0030925 (PMC3267759; doi:10.1371/journal.pone.0030925)

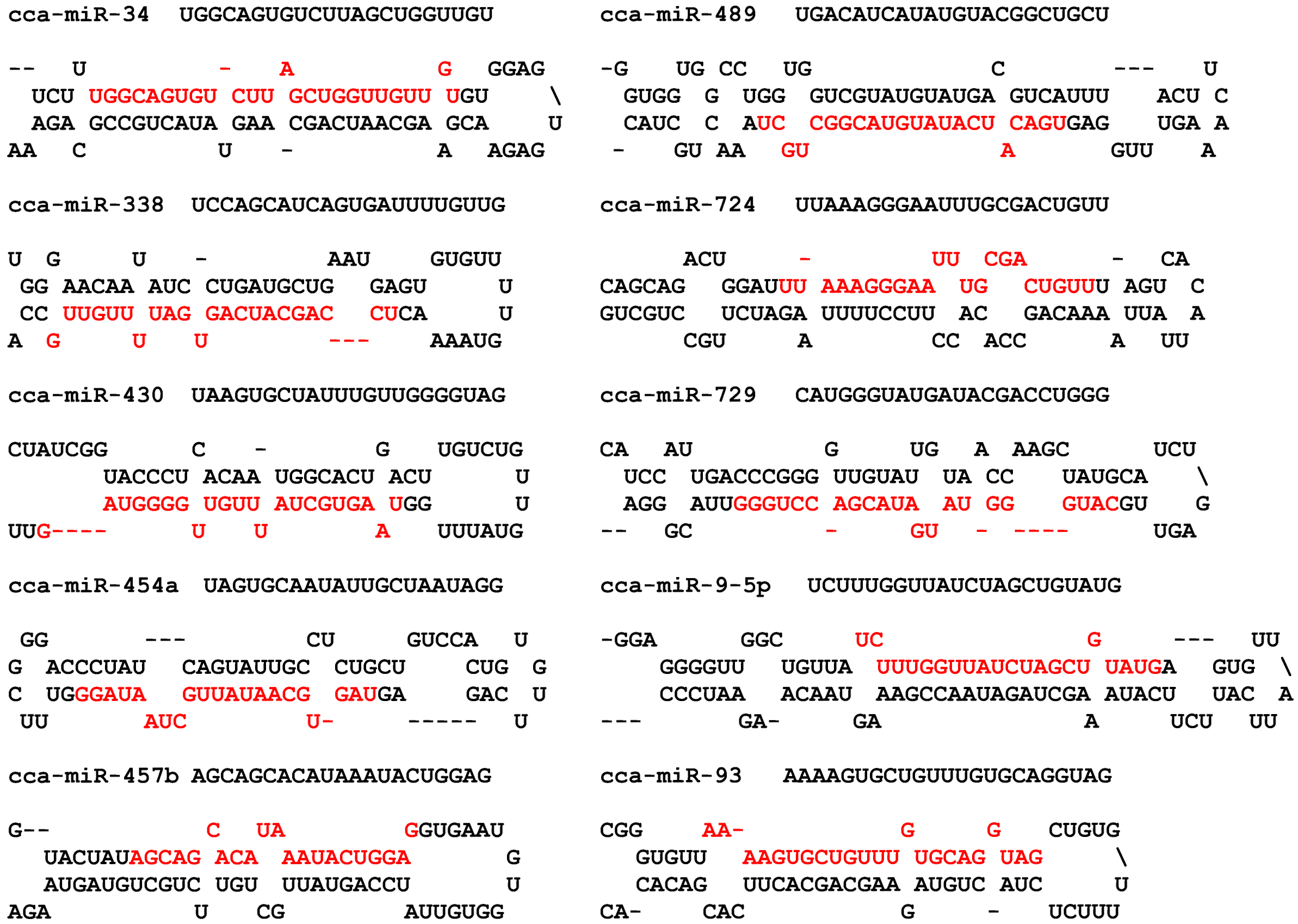

Supplement: Figure S1 — Prediction of the fold-back structure of 10 conserved carp miRNA precursors. The precursor sequences were obtained by sequence alignment with the sequences of the common carp genome and ESTs. The mature miRNA sequences in the precursors are indicated in red. (TIF) [file pone.0030925.s001.tif]

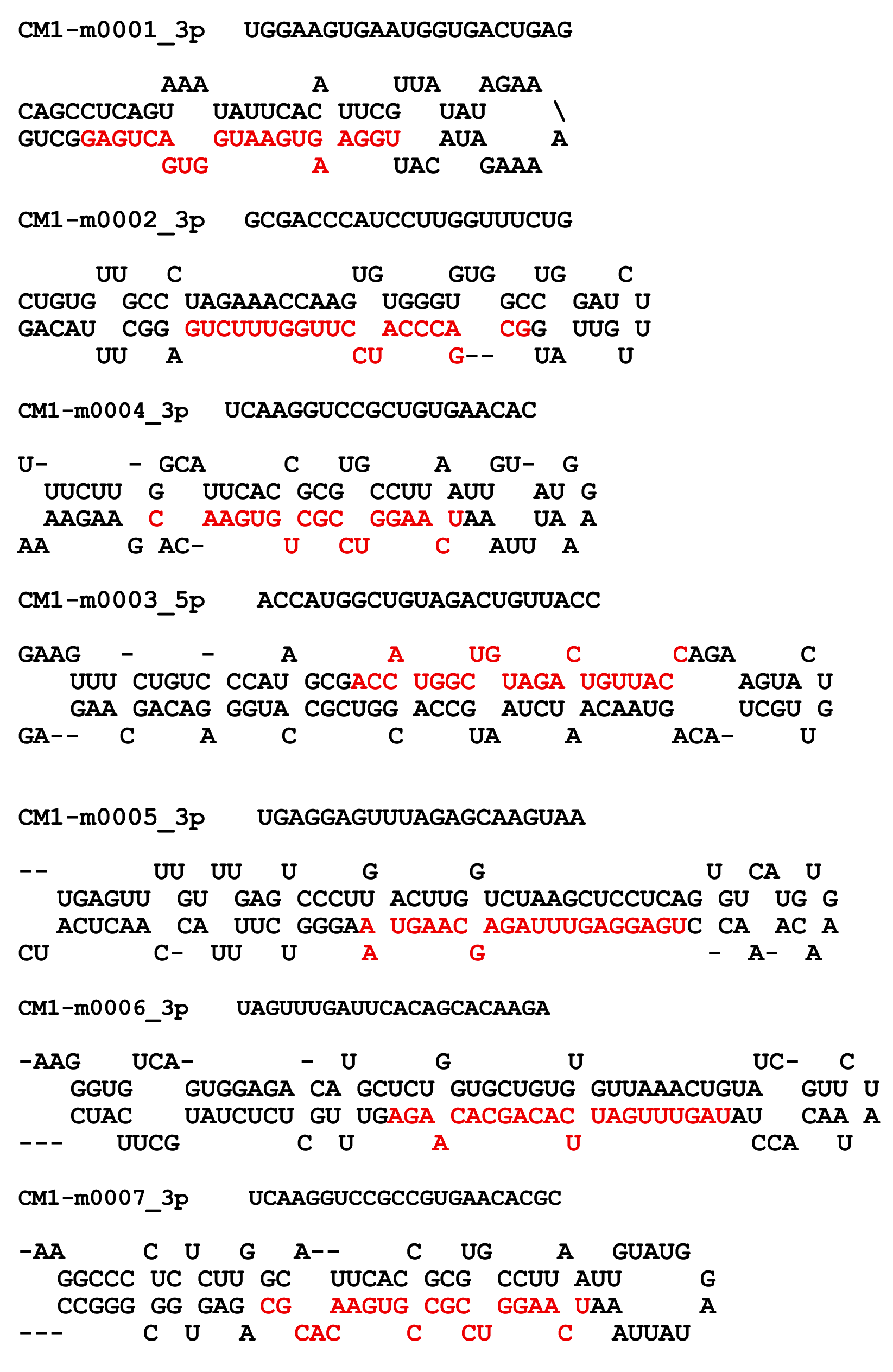

Supplement: Figure S2 — Prediction of the precursor structure of 7 novel carp miRNAs. The precursor sequences were obtained by sequence alignment with the sequences of the common carp genome and ESTs. The mature miRNA sequences in the precursors are indicated in red. (TIF) [file pone.0030925.s002.tif]

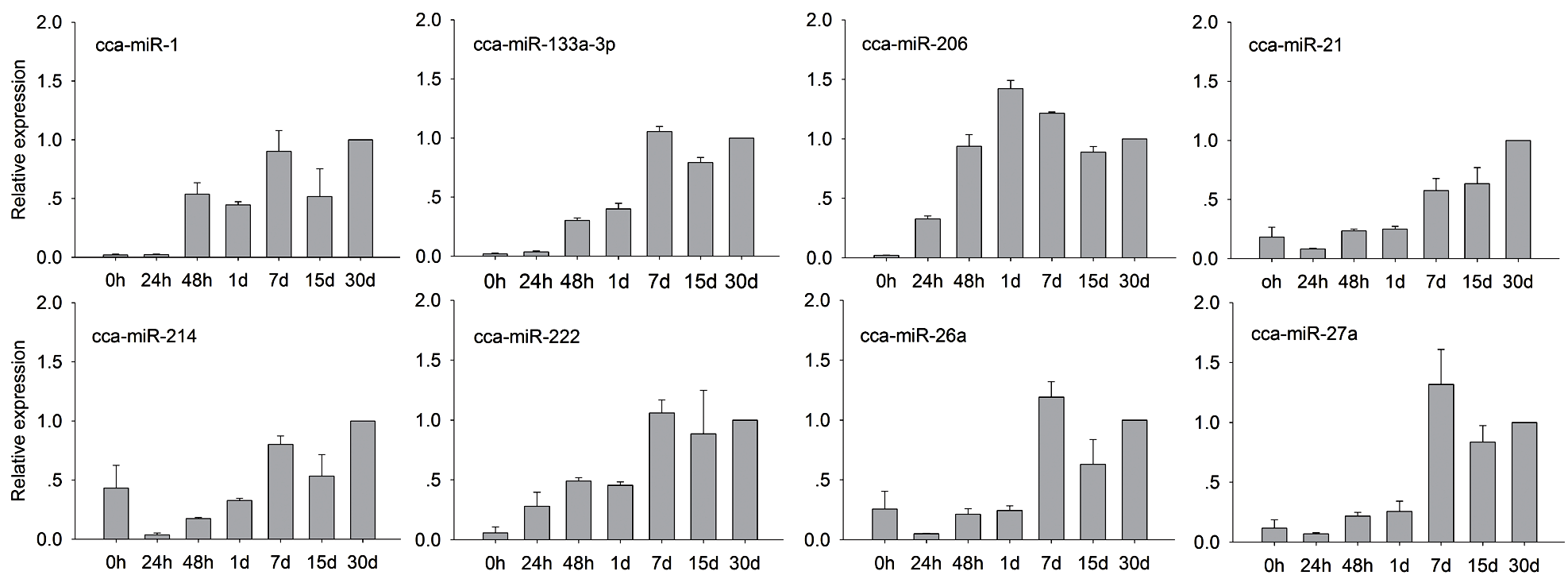

Supplement: Figure S3 — The expression of muscle-related miRNAs during development of the common carp was detected by qRT-PCR. 0 h, fertilized oocytes; 24 h, 24 hpf embryos; 48 h, 48 hpf embryos; 1 d, 1 dph larva; 7 d, 7 dph larva; 15 d, 15 dph larva; 30 d, 30 dph larva. At least 5 animals were used for each timepoint. The level of miRNA expression in the 30 dph larva was defined as 1. (TIF) [file pone.0030925.s003.tif]
