# Supplementary material for: Identification and Profiling of MicroRNAs from Skeletal Muscle of the Common Carp
Source: PLoS One. 2012 Jan 27;7(1):e30925. doi: 10.1371/journal.pone.0030925 (PMC3267759; doi:10.1371/journal.pone.0030925)
Supplement: Table S1 — Highly-conserved miRNAs identified in the common carp. (DOC) [file pone.0030925.s004.doc]

**Table S1.** Highly-conserved miRNAs identified in the common carp.

| miRNA | Sequence | Frequency | Conserved in the model animal |
| --- | --- | --- | --- |
| cca-let-7a | UGAGGUAGUAGGUUGUAUAGUU | 319175 | cel dme dre xtr gga mmu hsa |
| cca-let-7b | UGAGGUAGUAGGUUGUGUGGUU | 122633 | dre xtr gga mmu hsa |
| cca-let-7c | UGAGGUAGUAGGUUGUAUGGUU | 85734 | dre xtr gga mmu hsa |
| cca-let-7d | UGAGGUAGUUGGUUGUAUGGUU | 67112 | dre gga mmu hsa |
| cca-let-7e | UGAGGUAGUAGAUUGAAUAGUU | 55679 | dre xtr mmu hsa |
| cca-let-7f | UGAGGUAGUAGAUUGUAUAGUU | 15924 | dre xtr gga mmu hsa |
| cca-let-7g | UGAGGUAGUAGUUUGUAUAGUU | 912 | dre xtr gga mmu hsa |
| cca-let-7h | UGAGGUAGUAAGUUGUGUUGU | 159 | dre |
| cca-let-7i | UGAGGUAGUAGUUUGUGCUGU | 510 | dre xtr gga mmu hsa |
| cca-let-7j | UGAGGUAGUUGUUUGUACAGUU | 72 | dre gga |
| cca-miR-1 | UGGAAUGUAAAGAAGUAUGUAU | 1850549 | cel dme dre xtr gga mmu hsa |
| cca-miR-100 | AACCCGUAGAUCCGAACUUGU | 12003 | dme dre xtr gga mmu hsa |
| cca-miR-101a | UACAGUACUGUGAUAACUGAAG | 18390 | dre xtr gga mmu hsa |
| cca-miR-101b | GUACAGUACUAUGAUAACUGA | 154 | dre mmu |
| cca-miR-103 | AGCAGCAUUGUACAGGGCUAUGA | 3806 | dre xtr gga mmu hsa |
| cca-miR-107 | AGCAGCAUUGUACAGGGCUAUC | 18 | dre xtr gga mmu hsa |
| cca-miR-10a-5p | UACCCUGUAGAUCCGAAUUUGU | 4589 | dme dre xtr gga mmu hsa |
| cca-miR-10b | UACCCUGUAGAACCGAAUUUGU | 12421 | dre xtr gga mmu hsa |
| cca-miR-10c | UACCCUGUAGAUCCGGAUUUGUG | 2018 | dre xtr |
| cca-miR-10d | UACCCUGUAGAACCGAAUGUGU | 10 | dre |
| cca-miR-122 | UGGAGUGUGACAAUGGUGUUUG | 3445 | dre xtr gga mmu hsa |
| cca-miR-125a | UCCCUGAGACCCUUAACCUGUG | 220 | dme dre xtr mmu hsa |
| cca-miR-125b | UCCCUGAGACCCUAACUUGUGA | 6170 | dre xtr gga mmu hsa |
| cca-miR-125c | UCCCUGAGACCCUAACUCGUGA | 7625 | dre |
| cca-miR-126-3p | CUCGUACCGUGAGUAAUAAUGC | 4344 | dre xtr gga mmu hsa |
| cca-miR-128 | UCACAGUGAACCGGUCUCUUU | 120 | dre xtr gga mmu hsa |
| cca-miR-129 | CUUUUUGCGGUCUGGGCUUGC | 34 | dre xtr mmu hsa |
| cca-miR-130a | CAGUGCAAUGUUAAAAGGGCAU | 16 | dre xtr gga mmu hsa |
| cca-miR-130b | CAGUGCAAUAAUGAAAGGGCAU | 780 | dre xtr gga mmu hsa |
| cca-miR-130c | CAGUGCAAUAUUAAAAGGGCAU | 87 | dre xtr gga |
| cca-miR-132 | UAACAGUCUACAGCCAUGGUCG | 161 | dre xtr mmu hsa |
| cca-miR-133a-3p | UUGGUCCCCUUCAACCAGCUGU | 1506 | dme dre xtr gga mmu hsa |
| cca-miR-133b-3p | UUUGGUCCCCUUCAACCAGCUA | 13 | dre xtr gga mmu hsa |
| cca-miR-133c | UUUGGUCCCUUUCAACCAGCU | 10 | dre xtr gga |
| cca-miR-135a | UAUGGCUUUUUAUUCCUAUGUGA | 1 | dre xtr gga mmu hsa |
| cca-miR-135b | UAUGGCUUUUUAUUCCUAUCUGA | 9 | dre xtr gga mmu hsa |
| cca-miR-135c | UAUGGCUUUCUAUUCCUAUGUGA | 7 | dre |
| cca-miR-137 | UUAUUGCUUAAGAAUACGCGUAG | 28 | dme dre xtr gga mmu hsa |
| cca-miR-138 | AGCUGGUGUUGUGAAUCAGGC | 36 | dre xtr gga mmu hsa |
| cca-miR-139 | UCUACAGUGCAUGUGUCUCCAGU | 20 | cel dme dre xtr gga mmu hsa |
| cca-miR-140-5p | CAGUGGUUUUACCCUAUGGUAG | 356 | dre xtr mmu hsa |
| cca-miR-141 | UAACACUGUCUGGUAACGAUGC | 581 | dre mmu hsa |
| cca-miR-142a-3p | GUAGUGUUUCCUACUUUAUGG | 2047 | dre xtr gga mmu hsa |
| cca-miR-142a-5p | CAUAAAGUAGAAAGCACUACU | 87 | dre xtr gga mmu hsa |
| cca-miR-142b-5p | CAUAAAGUAGACAGCACUACU | 8 | dre |
| cca-miR-143 | UGAGAUGAAGCACUGUAGCU | 42491 | dre xtr mmu hsa |
| cca-miR-144 | CUACAGUAUAGAUGAUGUACU | 65 | dre xtr gga mmu hsa |
| cca-miR-145 | GUCCAGUUUUCCCAGGAAUCCC | 21 | dre xtr mmu hsa |
| cca-miR-146a | UGAGAACUGAAUUCCAUAGAUGG | 93424 | dre xtr gga mmu hsa |
| cca-miR-146b | UGAGAACUGAAUUCCAAGGGUG | 1033 | dre xtr gga mmu hsa |
| cca-miR-148 | UCAGUGCAUUACAGAACUUUGU | 4743 | dre xtr gga mmu hsa |
| cca-miR-150 | UCUCCCAAUCCUUGUACCAGUG | 498 | dre xtr mmu hsa |
| cca-miR-152 | UCAGUGCAUGACAGAACUUUG | 1680 | dre mmu hsa |
| cca-miR-153b | UUGCAUAGUCACAAAAAUGAGC | 6 | dre |
| cca-miR-153c | UUGCAUAGUCACAAAAAUGAUC | 2 | dre |
| cca-miR-155 | UUAAUGCUAAUCGUGAUAGGGG | 109 | dre xtr gga mmu hsa |
| cca-miR-15a-5p | UAGCAGCACAGAAUGGUUUGU | 179 | dre xtr gga mmu hsa |
| cca-miR-15b | UAGCAGCACAUCAUGGUUUGUA | 36 | dre xtr gga mmu hsa |
| cca-miR-16a | UAGCAGCACGUAAAUAUUGGUG | 16 | dre xtr gga mmu hsa |
| cca-miR-16b | UAGCAGCACGUAAAUAUUGGAG | 3871 | dre xtr |
| cca-miR-16c | UAGCAGCAUGUAAAUAUUGGA | 245 | dre xtr gga |
| cca-miR-17-5p | CAAAGUGCUUACAGUGCAGGUAG | 1354 | dre xtr gga mmu hsa |
| cca-miR-181a-5p | AACAUUCAACGCUGUCGGUGA | 14161 | dre xtr gga mmu hsa |
| cca-miR-181b | AACAUUCAUUGCUGUCGGUGG | 2484 | dre xtr gga mmu hsa |
| cca-miR-181c | CACAUUCAUUGCUGUCGGUGGG | 93 | dre |
| cca-miR-182-5p | UUUGGCAAUGGUAGAACUCACAC | 835 | dre xtr mmu hsa |
| cca-miR-183 | UAUGGCACUGGUAGAAUUCACUG | 1869 | dre xtr gga mmu hsa |
| cca-miR-184 | UGGACGGAGAACUGAUAAGGGC | 1031 | dme dre xtr gga mmu hsa |
| cca-miR-187 | UCGUGUCUUGUGUUGCAGCCAGU | 38 | dre xtr gga mmu hsa |
| cca-miR-18a | UAAGGUGCAUCUAGUGCAGAUAG | 259 | dre xtr gga mmu hsa |
| cca-miR-18b | UAAGGUGCAUUUAGUGCAGAUAG | 3 | dre xtr gga mmu hsa |
| cca-miR-18c | UAAGGUGCAUCUUGUGUAGUUAG | 64 | dre |
| cca-miR-190 | UGAUAUGUUUGAUAUAUUAGG | 28 | dme dre gga mmu hsa |
| cca-miR-192 | AUGACCUAUGAAUUGACAGCC | 8332 | dre xtr mmu hsa |
| cca-miR-193a | AACUGGCCUACAAAGUCCCAGU | 7 | dme dre xtr gga mmu hsa |
| cca-miR-193b | AACUGGCCCGCAAAGUCCCGCU | 36 | dre gga mmu hsa |
| cca-miR-194 | UGUAACAGCAACUCCAUGUGGA | 188 | dre xtr gga mmu hsa |
| cca-miR-196a | UAGGUAGUUUCAUGUUGUUGGG | 12343 | dre xtr gga mmu hsa |
| cca-miR-196b | UAGGUAGUUUCAAGUUGUUGGG | 10453 | dre xtr mmu hsa |
| cca-miR-199-5p | CCCAGUGUUCAGACUACCUGUUC | 1133 | dre xtr gga mmu hsa |
| cca-miR-19a | UGUGCAAAUCUAUGCAAAACUGA | 49 | dre xtr gga mmu hsa |
| cca-miR-19b-3p | UGUGCAAAUCCAUGCAAAACUGA | 336 | dre xtr gga mmu hsa |
| cca-miR-19c | UGUGCAAAUCCAUGCAAAACUCG | 12 | dre |
| cca-miR-19d | UGUGCAAACCCAUGCAAAACUGA | 59 | dre |
| cca-miR-200a | UAACACUGUCUGGUAACGAUG | 72 | dre mmu hsa |
| cca-miR-200b | UAAUACUGCCUGGUAAUGAUGA | 50 | dre xtr gga mmu hsa |
| cca-miR-200c | UAAUACUGCCUGGUAAUGAUGC | 88 | dre mmu hsa |
| cca-miR-203a | GUGAAAUGUUUAGGACCACUUG | 80 | dre xtr gga mmu hsa |
| cca-miR-203b-3p | GUGAAAUGUUCAGGACCACUUG | 363 | dre |
| cca-miR-204 | UUCCCUUUGUCAUCCUAUGCCU | 25 | dre xtr gga mmu hsa |
| cca-miR-205 | UCCUUCAUUCCACCGGAGUCUG | 17 | dre xtr gga mmu hsa |
| cca-miR-206 | UGGAAUGUAAGGAAGUGUGUGG | 7555941 | dre xtr gga mmu hsa |
| cca-miR-20a-5p | UAAAGUGCUUAUAGUGCAGGUAG | 3474 | dre xtr gga mmu hsa |
| cca-miR-20b | CAAAGUGCUCACAGUGCAGGUAG | 101 | dre gga mmu hsa |
| cca-miR-21 | UAGCUUAUCAGACUGGUGUUGGC | 2082419 | dre gga mmu hsa |
| cca-miR-210-3p | CUGUGCGUGUGACAGCGGCU | 147 | dme dre xtr mmu hsa |
| cca-miR-214 | UACAGCAGGCACAGACAGG | 267 | dre xtr gga mmu hsa |
| cca-miR-216a | UAAUCUCAGCUGGCAACUGUGA | 568 | dre xtr gga mmu hsa |
| cca-miR-216b | UAAUCUCUGCAGGCAACUGUGA | 133 | dre gga mmu hsa |
| cca-miR-217 | UACUGCAUCAGGAACUGAUUGGA | 2516 | dre xtr gga mmu hsa |
| cca-miR-218a | UUGUGCUUGAUCUAACCAUGUG | 65 | dre xtr gga mmu hsa |
| cca-miR-218b | UUGUGCUUGAUCUAACCAUGC | 2 | dre |
| cca-miR-221 | AGCUACAUUGUCUGCUGGG | 26 | dre xtr gga mmu hsa |
| cca-miR-222 | AGCUACAUCUGGCUACUGGG | 45 | dre xtr gga mmu hsa |
| cca-miR-223 | UGUCAGUUUGUCAAAUACCCCA | 29 | dre xtr gga mmu hsa |
| cca-miR-22a | AAGCUGCCAGCUGAAGAACUGU | 25435 | dre xtr gga mmu hsa |
| cca-miR-22b | AAGCUGCCAGUUGAAGAGCUGU | 80 | dre |
| cca-miR-23a | AUCACAUUGCCAGGGAUUUCC | 130 | dre xtr mmu hsa |
| cca-miR-23b | AUCACAUUGCCAGGGAUUACC | 45 | dre xtr gga mmu hsa |
| cca-miR-24 | UGGCUCAGUUCAGCAGGAACAG | 3027 | dre xtr gga mmu hsa |
| cca-miR-25 | CAUUGCACUUGUCUCGGUCUGA | 1055 | dre xtr mmu hsa |
| cca-miR-26a | UUCAAGUAAUCCAGGAUAGGCU | 8278 | dre xtr gga mmu hsa |
| cca-miR-26b | UUCAAGUAAUCCAGGAUAGGUU | 756 | dre mmu hsa |
| cca-miR-27a | UUCACAGUGGCUAAGUUCCGC | 558 | dre xtr mmu hsa |
| cca-miR-27b | UUCACAGUGGCUAAGUUCUGC | 2814 | dre xtr gga mmu hsa |
| cca-miR-27c-3p | UUCACAGUGGUUAAGUUCUGCC | 639 | dre xtr |
| cca-miR-27d | UUCACAGUGGCUAAGUUCUUC | 22 | dre |
| cca-miR-27e | UUCACAGUGGCUAAGUUCAGU | 126 | dre |
| cca-miR-29a | UAGCACCAUUUGAAAUCGGUUA | 13 | dre xtr gga mmu hsa |
| cca-miR-29b | UAGCACCAUUUGAAAUCAGUGUU | 129 | dre xtr gga mmu hsa |
| cca-miR-301a | CAGUGCAAUAGUAUUGUCAAAGC | 56 | dre xtr gga mmu hsa |
| cca-miR-301b | CAGUGCAAUAGUAUUGUCAUUGC | 4 | dre gga mmu hsa |
| cca-miR-301c | CAGUGCAAUAGUAUUGUCAUAGC | 7 | dre xtr |
| cca-miR-30a | UGUAAACAUUCCCGACUGGAAG | 17 | dre xtr gga mmu hsa |
| cca-miR-30b | UGUAAACAUCCUACACUCAGCU | 184 | dre xtr gga mmu hsa |
| cca-miR-30c | UGUAAACAUCCUACACUCUCAGC | 633 | dre xtr gga mmu hsa |
| cca-miR-30d | UGUAAACAUCCCCGACUGGAAGC | 24799 | dre xtr gga mmu hsa |
| cca-miR-30e-5p | UGUAAACAUCCUUGACUGGAAGC | 16020 | dre xtr gga mmu hsa |
| cca-miR-31 | UGGCAAGAUGUUGGCAUAGCUG | 79 | dme dre xtr gga mmu hsa |
| cca-miR-338 | UCCAGCAUCAGUGAUUUUGUUG | 755 | dre xtr mmu hsa |
| cca-miR-34 | UGGCAGUGUCUUAGCUGGUUGU | 7 | dme dre xtr gga mmu hsa |
| cca-miR-363 | AAUUGCACGGUAUCCAUCUGUA | 206 | dre xtr mmu hsa |
| cca-miR-365 | UAAUGCCCCUAAAAAUCCUUAU | 34 | dre xtr gga mmu hsa |
| cca-miR-375 | UUUGUUCGUUCGGCUCGCGUUA | 57 | dme dre xtr gga mmu hsa |
| cca-miR-429 | UAAUACUGUCUGGUAAUGCCGU | 32 | dre xtr gga mmu hsa |
| cca-miR-430 | UAAGUGCUAUUUGUUGGGGUAG | 2 | dre |
| cca-miR-451 | AAACCGUUACCAUUACUGAGU | 609 | dre xtr gga mmu hsa |
| cca-miR-454a | UAGUGCAAUAUUGCUAAUAGG | 15 | dre gga hsa |
| cca-miR-454b | UAGUGCAAUAUUGCUUAUAGG | 36 | dre |
| cca-miR-455 | UAUGUGCCCUUGGACUACAUCG | 62 | dre xtr gga mmu hsa |
| cca-miR-456 | CAGGCUGGUUAGAUGGUUGUCA | 367 | dre gga |
| cca-miR-457a | AGCAGCACAUCAAUAUUGGC | 4 | dre |
| cca-miR-457b | AGCAGCACAUAAAUACUGGAG | 1 | dre |
| cca-miR-458 | AUAGCUCUUUGAAUGGUACUGC | 9442 | dre gga |
| cca-miR-459-5p | AGUAACAAGGAUUCAUCCUGUU | 4 | dre |
| cca-miR-460-3p | CACAGCGCAUACAAUGUGGAUG | 7 | dre |
| cca-miR-460-5p | CCUGCAUUGUACACACUGUGCG | 28 | dre gga |
| cca-miR-462 | UAACGGAACCCAUAAUGCAGCUG | 1518 | dre |
| cca-miR-489 | UGACAUCAUAUGUACGGCUGCU | 201 | dre xtr gga mmu hsa |
| cca-miR-499 | UUAAGACUUGCAGUGAUGUUUA | 202 | dre xtr gga mmu hsa |
| cca-miR-722 | UUUUUUGCAGAAACGUUUCAG | 53 | dre |
| cca-miR-724 | UUAAAGGGAAUUUGCGACUGUU | 65 | dre |
| cca-miR-725 | UUCAGUCAUUGUUUCUAGUAGU | 58 | dre |
| cca-miR-726 | UUCACUACUAGCAGAACUCGG | 19 | dre |
| cca-miR-727-3p | GUUGAGGCGAGUUGAAGACUUA | 4 | dre |
| cca-miR-729 | CAUGGGUAUGAUACGACCUGGG | 1 | dre |
| cca-miR-730 | UCCUCAUUGUGCAUGCUGUGUG | 154 | dre |
| cca-miR-731 | AAUGACACGUUUUCUCCCGGAUC | 97 | dre |
| cca-miR-734 | UAAAUGCUGCAGAAUCGUACCG | 7 | dre |
| cca-miR-738 | GCUACGGCCCGCGUCGGGA | 1 | dre |
| cca-miR-7a | UGGAAGACUAGUGAUUUUGUUGUU | 55 | dme dre xtr gga mmu hsa |
| cca-miR-7b | UGGAAGACUUGUGAUUUUGUUGU | 18 | dre gga mmu |
| cca-miR-9-5p | UCUUUGGUUAUCUAGCUGUAUG | 240 | dme dre xtr gga mmu hsa |
| cca-miR-92a | UAUUGCACUUGUCCCGGCCUGU | 6107 | dme dre xtr gga mmu hsa |
| cca-miR-92b | UAUUGCACUCGUCCCGGCCUCC | 27 | dme dre xtr mmu hsa |
| cca-miR-93 | AAAAGUGCUGUUUGUGCAGGUAG | 1263 | dre xtr mmu hsa |
| cca-miR-96 | UUUGGCACUAGCACAUUUUUGCU | 63 | dre xtr mmu hsa |
| cca-miR-99 | AACCCGUAGAUCCGAUCUUGU | 552 | dre xtr gga mmu hsa |
| cca-miR-10a-3p | CAAAUUCGUGUCUUGGGGAAU | 4 | dre gga mmu hsa |
| cca-miR-126-5p | CAUUAUUACUUUUGGUACGCG | 547 | dre xtr gga mmu hsa |
| cca-miR-133a-5p | AGCUGGUAAAAUGGAACCAAA | 480 | dre mmu |
| cca-miR-133b-5p | UGGUCAAAUGGAACCAAGUCAG | 23 | dre mmu |
| cca-miR-140-3p | UACCACAGGGUAGAACCACGGA | 952 | dre gga mmu hsa |
| cca-miR-15a-3p | CAGGCCGUACUGUGCUGCGG | 10 | dre mmu hsa |
| cca-miR-17-3p | ACUGCAGUGGAGGCACUUCUAGC | 2 | dre xtr gga mmu hsa |
| cca-miR-181a-3p | ACCAUCGACCGUUGAUUGUACC | 56 | dre xtr gga mmu hsa |
| cca-miR-182-3p | GUGGUUCUAGACUUGCCAACUA | 2 | dre xtr mmu hsa |
| cca-miR-199-3p | ACAGUAGUCUGCACAUUGGUU | 2206 | dre xtr gga mmu hsa |
| cca-miR-19b-5p | AGUUUUGCUGGUUUGCAUUCAG | 3 | dre mmu hsa |
| cca-miR-202-5p | UUCCUAUGCAUAUACCUCUUUGA | 1 | dre xtr gga mmu hsa |
| cca-miR-203b-5p | AGUGGUUCUCAACAGUUCAACAG | 3 | dre mmu |
| cca-miR-20a-3p | ACUGCAGUGUGAGCACUUGAAGU | 3 | dre hsa |
| cca-miR-210-5p | AGCCACUGACUAACGCACAUUG | 296 | dre mmu |
| cca-miR-27c-5p | CAGGACUUAACCCACUUGUGAAC | 310 | dre |
| cca-miR-30e-3p | CUUUCAGUCGGAUGUUUGCAGC | 15930 | dre xtr gga mmu hsa |
| cca-miR-459-3p | CAGGGAAUCUCUGUUACUGGG | 4 | dre |
| cca-miR-727-5p | UCAGUCUUCAAUUCCUCCCAGC | 9 | dre |
| cca-miR-9-3p | UAAAGCUAGAUAACCGAAAGUA | 1 | dme dre xtr gga mmu hsa |

cel: *Caenorhabditis elegans*; dme: *Drosophila melanogaster*; dre: *Danio rerio*; xtr: *Xenopus tropicalis*; gga: *Gallus gallus*; mmu: *Mus musculus*; hsa: *Homo sapiens*;
