# Supplementary material for: Identification and Profiling of MicroRNAs from Skeletal Muscle of the Common Carp
Source: PLoS One. 2012 Jan 27;7(1):e30925. doi: 10.1371/journal.pone.0030925 (PMC3267759; doi:10.1371/journal.pone.0030925)
Supplement: Table S2 — miRNA families identified in the common carp. (DOC) [file pone.0030925.s005.doc]

**Table S2.** miRNA families identified in the common carp.

| **family** | **member** | **sequence** |
| --- | --- | --- |
| let-7 | cca-let-7a | UGAGGUAGUAGGUUGUAUAGUU |
|  | cca-let-7b | UGAGGUAGUAGGUUGU**G**U**G**GUU |
|  | cca-let-7c | UGAGGUAGUAGGUUGUAU**G**GUU |
|  | cca-let-7d | UGAGGUAGU**U**GGUUGUAU**G**GUU |
|  | cca-let-7e | UGAGGUAGUAG**A**UUG**A**AUAGUU |
|  | cca-let-7f | UGAGGUAGUAG**A**UUGUAUAGUU |
|  | cca-let-7g | UGAGGUAGUAG**U**UUGUAUAGUU |
|  | cca-let-7h | UGAGGUAGUA**A**GUUGU**G**U**U**GU |
|  | cca-let-7i | UGAGGUAGUAG**U**UUGU**GCU**GU |
|  | cca-let-7j | UGAGGUAGUUG**U**UUGUA**C**AGUU |
| miR-101 | cca-miR-101a | UACAGUACUGUGAUAACUGAAG |
|  | cca-miR-101b | GUACAGUACU**A**UGAUAACUGA |
| miR-10 | cca-miR-10a-5p | UACCCUGUAGAUCCGAAUUUGU |
|  | cca-miR-10b | UACCCUGUAGA**A**CCGAAUUUGU |
|  | cca-miR-10c | UACCCUGUAGAUCCG**G**AUUUGUG |
|  | cca-miR-10d | UACCCUGUAGA**A**CCGAAU**G**UGU |
| miR-125 | cca-miR-125a | UCCCUGAGACCCU**U**A**AC**C**UGUG** |
|  | cca-miR-125b | UCCCUGAGACCCUAACU**U**GUGA |
|  | cca-miR-125c | UCCCUGAGACCCUAACUCGUGA |
| miR-130 | cca-miR-130a | CAGUGCAAU**G**UUAAAAGGGCAU |
|  | cca-miR-130b | CAGUGCAAUA**A**U**G**AAAGGGCAU |
|  | cca-miR-130c | CAGUGCAAUAUUAAAAGGGCAU |
| miR-133 | cca-miR-133a-3p | UUGGUCCCCUUCAACCAGCUGU |
|  | cca-miR-133b-3p | UUUGGUCCCCUUCAACCAGCU**A** |
|  | cca-miR-133c | UUUGGUCCC**U**UUCAACCAGCU |
| miR-135 | cca-miR-135a | UAUGGCUUUUUAUUCCUAUGUGA |
|  | cca-miR-135b | UAUGGCUUUUUAUUCCUAU**C**UGA |
|  | cca-miR-135c | UAUGGCUUU**C**UAUUCCUAUGUGA |
| miR-142 | cca-miR-142a-5p | CAUAAAGUAGAAAGCACUACU |
|  | cca-miR-142b-5p | CAUAAAGUAGA**C**AGCACUACU |
| miR-146 | cca-miR-146a | UGAGAACUGAAUUCCAUAGAUGG |
|  | cca-miR-146b | UGAGAACUGAAUUCCA**AG**G**G**UG |
| miR-153 | cca-miR-153b | UUGCAUAGUCACAAAAAUGAGC |
|  | cca-miR-153c | UUGCAUAGUCACAAAAAUGA**U**C |
| miR-15 | cca-miR-15a-5p | UAGCAGCACAGAAUGGUUUGU |
|  | cca-miR-15b | UAGCAGCACAU**C**AUGGUUUGUA |
| miR-16 | cca-miR-16a | UAGCAGCACGUAAAUAUUGG**U**G |
|  | cca-miR-16b | UAGCAGCACGUAAAUAUUGGAG |
|  | cca-miR-16c | UAGCAGCA**U**GUAAAUAUUGGA |
| miR-181 | cca-miR-181a-5p | AACAUUCA**AC**GCUGUCGGUG**A** |
|  | cca-miR-181b | AACAUUCAUUGCUGUCGGUGG |
|  | cca-miR-181c | **C**ACAUUCAUUGCUGUCGGUGGG |
| miR-18 | cca-miR-18a | UAAGGUGCAUCUAGUGCAGAUAG |
|  | cca-miR-18b | UAAGGUGCAU**U**UAGUGCAGAUAG |
|  | cca-miR-18c | UAAGGUGCAUCU**U**GUG**U**AG**U**UAG |
| miR-193 | cca-miR-193a | AACUGGCCUACAAAGUCCCAGU |
|  | cca-miR-193b | AACUGGCC**CG**CAAAGUCCC**GC**U |
| miR-196 | cca-miR-196a | UAGGUAGUUUCAUGUUGUUGGG |
|  | cca-miR-196b | UAGGUAGUUUCA**A**GUUGUUGGG |
| miR-19 | cca-miR-19a | UGUGCAAAUCUAUGCAAAACUGA |
|  | cca-miR-19b-3p | UGUGCAAAUC**C**AUGCAAAACUGA |
|  | cca-miR-19c | UGUGCAAAUCCAUGCAAAACUCG |
|  | cca-miR-19d | UGUGCAAA**C**CCAUGCAAAACU**GA** |
| miR-200 | cca-miR-200a | UAA**C**ACUG**U**CUGGUAA**C**GAUG |
|  | cca-miR-200b | UAAUACUGCCUGGUAAUGAUGA |
|  | cca-miR-200c | UAAUACUGCCUGGUAAUGAUG**C** |
| miR-203 | cca-miR-203a | GUGAAAUGUUUAGGACCACUUG |
|  | cca-miR-203b-3p | GUGAAAUGUU**C**AGGACCACUUG |
| miR-20 | cca-miR-20a-5p | UAAAGUGCUUAUAGUGCAGGUAG |
|  | cca-miR-20b | CAAAGUGCU**C**A**C**AGUGCAGGUAG |
| miR-216 | cca-miR-216a | UAAUCUCAGCUGGCAACUGUGA |
|  | cca-miR-216b | UAAUCUC**U**GC**A**GGCAACUGUGA |
| miR-218 | cca-miR-218a | UUGUGCUUGAUCUAACCAUGUG |
|  | cca-miR-218b | UUGUGCUUGAUCUAACCAUG**C** |
| miR-22 | cca-miR-22a | AAGCUGCCAGCUGAAGAACUGU |
|  | cca-miR-22b | AAGCUGCCAG**U**UGAAGA**G**CUGU |
| miR-23 | cca-miR-23a | AUCACAUUGCCAGGGAUUUCC |
|  | cca-miR-23b | AUCACAUUGCCAGGGAUU**A**CC |
| miR-26 | cca-miR-26a | UUCAAGUAAUCCAGGAUAGGCU |
|  | cca-miR-26b | UUCAAGUAAUCCAGGAUAGG**U**U |
| miR-27 | cca-miR-27a | UUCACAGUGGCUAAGUUC**C**GC |
|  | cca-miR-27b | UUCACAGUGGCUAAGUUCUGC |
|  | cca-miR-27c-3p | UUCACAGUGG**U**UAAGUUCUGCC |
|  | cca-miR-27d | UUCACAGUGGCUAAGUUCU**U**C |
|  | cca-miR-27e | UUCACAGUGGCUAAGUUC**A**G**U** |
| miR-29 | cca-miR-29a | UAGCACCAUUUGAAAUCGGUUA |
|  | cca-miR-29b | UAGCACCAUUUGAAAUC**A**GU**GU**U |
| miR-301 | cca-miR-301a | CAGUGCAAUAGUAUUGUCA**A**AGC |
|  | cca-miR-301b | CAGUGCAAUAGUAUUGUCAU**U**GC |
|  | cca-miR-301c | CAGUGCAAUAGUAUUGUCAUAGC |
| miR-30 | cca-miR-30a | UGUAAACAU**U**CCCGACUGGAAG |
|  | cca-miR-30b | UGUAAACAUCC**UAC**ACU**CAGCU** |
|  | cca-miR-30c | UGUAAACAUCC**UAC**ACU**CUC**AGC |
|  | cca-miR-30d | UGUAAACAUCCCCGACUGGAAGC |
|  | cca-miR-30e-5p | UGUAAACAUCC**UU**GACUGGAAGC |
| miR-454 | cca-miR-454a | UAGUGCAAUAUUGCUAAUAGG |
|  | cca-miR-454b | UAGUGCAAUAUUGCU**U**AUAGG |
| miR-457 | cca-miR-457a | AGCAGCACAUCAAUAUUGGC |
|  | cca-miR-457b | AGCAGCACAU**A**AAUA**C**UGG**A**G |
| miR-7 | cca-miR-7a | UGGAAGACUAGUGAUUUUGUUGUU |
|  | cca-miR-7b | UGGAAGACU**U**GUGAUUUUGUUGU |
| miR-92 | cca-miR-92a | UAUUGCACUUGUCCCGGCCUGU |
|  | cca-miR-92b | UAUUGCACU**C**GUCCCGGCCU**CC** |

Bases that differ between family members are indicated in bold.
