# Supplementary material for: Identification and Profiling of MicroRNAs from Skeletal Muscle of the Common Carp
Source: PLoS One. 2012 Jan 27;7(1):e30925. doi: 10.1371/journal.pone.0030925 (PMC3267759; doi:10.1371/journal.pone.0030925)
Supplement: Table S3 — Comparison between the discovered miRNAs in the common carp and those found in the bighead carp and silver carp. (DOC) [file pone.0030925.s006.doc]

**Table S3. Comparison between the discovered miRNAs in the common carp and those found in the bighead carp and s**ilver carp.

| **Common carp miRNA** | **sequence** | **Bighead carp miRNA** | **sequence** | **Silver carp miRNA** | **sequence** |
| --- | --- | --- | --- | --- | --- |
| cca-let-7a | UGAGGUAGUAGGUUGUAUAGUU | hno-let-7a | UGAGGUAGUAGGUUGUAUAGUU | hmo-let-7a | UGAGGUAGUAGGUUGUAUAGUU |
| cca-let-7b | UGAGGUAGUAGGUUGUGUGGUU | hno-let-7b | UGAGGUAGUAGGUUGUGUGGUU | hmo-let-7b | UGAGGUAGUAGGUUGUGUGGUU |
| cca-let-7c | UGAGGUAGUAGGUUGUAUGGUU | hno-let-7c | UGAGGUAGUAGGUUGUAUGGUU | hmo-let-7c | UGAGGUAGUAGGUUGUAUGGUU |
| cca-let-7d | UGAGGUAGUUGGUUGUAUGGUU | hno-let-7d | UGAGGUAGUUGGUUGUAUGGUU | hmo-let-7d | UGAGGUAGUUGGUUGUAUGGUU |
| cca-let-7e | UGAGGUAGUAGAUUGAAUAGUU | hno-let-7e | UGAGGUAGUAGAUUGAAUAGUU | hmo-let-7e | UGAGGUAGUAGAUUGAAUAGUU |
| cca-let-7f | UGAGGUAGUAGAUUGUAUAGUU | hno-let-7f | UGAGGUAGUAGAUUGUAUAGUU | hmo-let-7f | UGAGGUAGUAGAUUGUAUAGUU |
| cca-let-7g | UGAGGUAGUAGUUUGUAUAGUU | hno-let-7g | UGAGGUAGUAGUUUGUAUAGUU | hmo-let-7g | UGAGGUAGUAGUUUGUAUAGUU |
| cca-let-7h | UGAGGUAGUAAGUUGUGUUGU | hno-let-7h | UGAGGUAGUAAGUUGUGUUGUU | hmo-let-7h | UGAGGUAGUAAGUUGUGUUGUU |
| cca-let-7i | UGAGGUAGUAGUUUGUGCUGU | hno-let-7i | UGAGGUAGUAGUUUGUGCUGUU | hmo-let-7i | UGAGGUAGUAGUUUGUGCUGUU |
| cca-let-7j | UGAGGUAGUUGUUUGUACAGUU | hno-let-7j | UGAGGUAGUUGUUUGUACAGUU | hmo-let-7j | UGAGGUAGUUGUUUGUACAGUU |
| cca-miR-1 | UGGAAUGUAAAGAAGUAUGUAU | hno-miR-1 | UGGAAUGUAAAGAAGUAUGUAU | hmo-miR-1 | UGGAAUGUAAAGAAGUAUGUAU |
| cca-miR-100 | AACCCGUAGAUCCGAACUUGU | hno-miR-100 | AACCCGUAGAUCCGAACUUGUG | hmo-miR-100 | AACCCGUAGAUCCGAACUUGUG |
| cca-miR-101a | UACAGUACUGUGAUAACUGAAG | hno-miR-101a | UACAGUACUGUGAUAACUGAAG | hmo-miR-101a | UACAGUACUGUGAUAACUGAAG |
| cca-miR-101b | GUACAGUACUAUGAUAACUGA | hno-miR-101b | UACAGUACUAUGAUAACUGAAG | hmo-miR-101b | UACAGUACUAUGAUAACUGAAG |
| cca-miR-103 | AGCAGCAUUGUACAGGGCUAUGA | hno-miR-103 | AGCAGCAUUGUACAGGGCUAUGA | hmo-miR-103 | AGCAGCAUUGUACAGGGCUAUGA |
| cca-miR-107 | AGCAGCAUUGUACAGGGCUAUC | hno-miR-107 | AGCAGCAUUGUACAGGGCUAUCA | hmo-miR-107 | AGCAGCAUUGUACAGGGCUAUCA |
| cca-miR-10a-5p | UACCCUGUAGAUCCGAAUUUGU | hno-miR-10a | UACCCUGUAGAUCCGAAUUUGU | hmo-miR-10a | UACCCUGUAGAUCCGAAUUUGU |
| cca-miR-10b | UACCCUGUAGAACCGAAUUUGU | hno-miR-10b | UACCCUGUAGAACCGAAUUUGUG | hmo-miR-10b | UACCCUGUAGAACCGAAUUUGUG |
| cca-miR-10c | UACCCUGUAGAUCCGGAUUUGUG | hno-miR-10c | UACCCUGUAGAUCCGGAUUUGU | hmo-miR-10c | UACCCUGUAGAUCCGGAUUUGU |
| cca-miR-10d | UACCCUGUAGAACCGAAUGUGU | hno-miR-10d | UACCCUGUAGAACCGAAUGUGUG | hmo-miR-10d | UACCCUGUAGAACCGAAUGUGUG |
| cca-miR-122 | UGGAGUGUGACAAUGGUGUUUG | hno-miR-122 | UGGAGUGUGACAAUGGUGUUUG | hmo-miR-122 | UGGAGUGUGACAAUGGUGUUUG |
|  |  | hno-miR-124 | UAAGGCACGCGGUGAAUGCCAA | hmo-miR-124 | UAAGGCACGCGGUGAAUGCCAA |
| cca-miR-125a | UCCCUGAGACCCUUAACCUGUG | hno-miR-125a | UCCCUGAGACCCUUAACCUGUG | hmo-miR-125a | UCCCUGAGACCCUUAACCUGUG |
| cca-miR-125b | UCCCUGAGACCCUAACUUGUGA | hno-miR-125b | UCCCUGAGACCCUAACUUGUGA | hmo-miR-125b | UCCCUGAGACCCUAACUUGUGA |
| cca-miR-125c | UCCCUGAGACCCUAACUCGUGA | hno-miR-125c | UCCCUGAGACCCUAACUCGUGA | hmo-miR-125c | UCCCUGAGACCCUAACUCGUGA |
| cca-miR-126-3p | CUCGUACCGUGAGUAAUAAUGC | hno-miR-126 | UCGUACCGUGAGUAAUAAUGC | hmo-miR-126 | UCGUACCGUGAGUAAUAAUGC |
|  |  | hno-miR-126b | UCGUACCGUGAGUAAUAGUGCA | hmo-miR-126b | UCGUACCGUGAGUAAUAGUGCA |
| cca-miR-128 | UCACAGUGAACCGGUCUCUUU | hno-miR-128 | UCACAGUGAACCGGUCUCUUUU | hmo-miR-128 | UCACAGUGAACCGGUCUCUUUU |
| cca-miR-129 | CUUUUUGCGGUCUGGGCUUGC | hno-miR-129 | CUUUUUGCGGUCUGGGCUUGCU | hmo-miR-129 | CUUUUUGCGGUCUGGGCUUGCU |
| cca-miR-130a | CAGUGCAAUGUUAAAAGGGCAU | hno-miR-130a | CAGUGCAAUGUUAAAAGGGCAU | hmo-miR-130a | CAGUGCAAUGUUAAAAGGGCAU |
| cca-miR-130b | CAGUGCAAUAAUGAAAGGGCAU | hno-miR-130b | CAGUGCAAUAAUGAAAGGGCAU | hmo-miR-130b | CAGUGCAAUAAUGAAAGGGCAU |
| cca-miR-130c | CAGUGCAAUAUUAAAAGGGCAU | hno-miR-130c | CAGUGCAAUAUUAAAAGGGCAU | hmo-miR-130c | CAGUGCAAUAUUAAAAGGGCAU |
| cca-miR-132 | UAACAGUCUACAGCCAUGGUCG | hno-miR-132 | UAACAGUCUACAGCCAUGGUCG | hmo-miR-132 | UAACAGUCUACAGCCAUGGUCG |
| cca-miR-133a-3p | UUGGUCCCCUUCAACCAGCUGU | hno-miR-133a | UUUGGUCCCCUUCAACCAGCUG | hmo-miR-133a | UUUGGUCCCCUUCAACCAGCUG |
| cca-miR-133b-3p | UUUGGUCCCCUUCAACCAGCUA | hno-miR-133b | UUUGGUCCCCUUCAACCAGCUA | hmo-miR-133b | UUUGGUCCCCUUCAACCAGCUA |
| cca-miR-133c | UUUGGUCCCUUUCAACCAGCU | hno-miR-133c | UUUGGUCCCUUUCAACCAGCUA | hmo-miR-133c |  |
| cca-miR-135a | UAUGGCUUUUUAUUCCUAUGUGA | hno-miR-135a | UAUGGCUUUUUAUUCCUAUGUGA | hmo-miR-135a | UAUGGCUUUUUAUUCCUAUGUGA |
| cca-miR-135b | UAUGGCUUUUUAUUCCUAUCUGA | hno-miR-135b | UAUGGCUUUUUAUUCCUAUCUG | hmo-miR-135b | UAUGGCUUUUUAUUCCUAUCUG |
| cca-miR-135c | UAUGGCUUUCUAUUCCUAUGUGA | hno-miR-135c | UAUGGCUUUCUAUUCCUAUGUG | hmo-miR-135c | UAUGGCUUUCUAUUCCUAUGUG |
| cca-miR-137 | UUAUUGCUUAAGAAUACGCGUAG | hno-miR-137 | UUAUUGCUUAAGAAUACGCGUA | hmo-miR-137 | UUAUUGCUUAAGAAUACGCGUA |
| cca-miR-138 | AGCUGGUGUUGUGAAUCAGGC | hno-miR-138 | AGCUGGUGUUGUGAAUCAGGCC | hmo-miR-138 | AGCUGGUGUUGUGAAUCAGGCC |
| cca-miR-139 | UCUACAGUGCAUGUGUCUCCAGU |  |  |  |  |
|  |  | hno-miR-1388 | AUCUCAGGUUCGUCAGCCCAUG | hmo-miR-1388 | AUCUCAGGUUCGUCAGCCCAUG |
| cca-miR-140-5p | CAGUGGUUUUACCCUAUGGUAG | hno-miR-140 | CAGUGGUUUUACCCUAUGGUAG | hmo-miR-140 | CAGUGGUUUUACCCUAUGGUAG |
| cca-miR-141 | UAACACUGUCUGGUAACGAUGC | hno-miR-141 | UAACACUGUCUGGUAACGAUGC | hmo-miR-141 | UAACACUGUCUGGUAACGAUGC |
| cca-miR-142a-5p | CAUAAAGUAGAAAGCACUACU | hno-miR-142a | CAUAAAGUAGAAAGCACUACU | hmo-miR-142a | CAUAAAGUAGAAAGCACUACU |
| cca-miR-142b-5p | CAUAAAGUAGACAGCACUACU | hno-miR-142b | CAUAAAGUAGACAGCACUACUA | hmo-miR-142b | CAUAAAGUAGACAGCACUACUA |
| cca-miR-143 | UGAGAUGAAGCACUGUAGCU | hno-miR-143 | UGAGAUGAAGCACUGUAGCUC | hmo-miR-143 | UGAGAUGAAGCACUGUAGCUC |
| cca-miR-144 | CUACAGUAUAGAUGAUGUACU | hno-miR-144 | UACAGUAUAGAUGAUGUACU | hmo-miR-144 | UACAGUAUAGAUGAUGUACU |
| cca-miR-145 | GUCCAGUUUUCCCAGGAAUCCC | hno-miR-145 | GUCCAGUUUUCCCAGGAAUCCC | hmo-miR-145 | GUCCAGUUUUCCCAGGAAUCCC |
| cca-miR-146a | UGAGAACUGAAUUCCAUAGAUGG | hno-miR-146a | UGAGAACUGAAUUCCAUAGAUGG | hmo-miR-146a | UGAGAACUGAAUUCCAUAGAUGG |
| cca-miR-146b | UGAGAACUGAAUUCCAAGGGUG | hno-miR-146b | UGAGAACUGAAUUCCAAGGGUG | hmo-miR-146b | UGAGAACUGAAUUCCAAGGGUG |
| cca-miR-148 | UCAGUGCAUUACAGAACUUUGU | hno-miR-148 | UCAGUGCAUUACAGAACUUUGU | hmo-miR-148 | UCAGUGCAUUACAGAACUUUGU |
| cca-miR-150 | UCUCCCAAUCCUUGUACCAGUG | hno-miR-150 | UCUCCCAAUCCUUGUACCAGUG | hmo-miR-150 | UCUCCCAAUCCUUGUACCAGUG |
| cca-miR-152 | UCAGUGCAUGACAGAACUUUG | hno-miR-152 | UCAGUGCAUGACAGAACUUUGG | hmo-miR-152 | UCAGUGCAUGACAGAACUUUGG |
|  |  | hno-miR-153a | UUGCAUAGUCACAAAAGUGAUC | hmo-miR-153a | UUGCAUAGUCACAAAAGUGAUC |
| cca-miR-153b | UUGCAUAGUCACAAAAAUGAGC | hno-miR-153b | UUGCAUAGUCACAAAAAUGAGC | hmo-miR-153b | UUGCAUAGUCACAAAAAUGAGC |
| cca-miR-153c | UUGCAUAGUCACAAAAAUGAUC | hno-miR-153c | UUGCAUAGUCACAAAAAUGAUC | hmo-miR-153c | UUGCAUAGUCACAAAAAUGAUC |
| cca-miR-155 | UUAAUGCUAAUCGUGAUAGGGG | hno-miR-155 | UUAAUGCUAAUCGUGAUAGGGG | hmo-miR-155 | UUAAUGCUAAUCGUGAUAGGGG |
| cca-miR-15a-5p | UAGCAGCACAGAAUGGUUUGU | hno-miR-15a | UAGCAGCACAGAAUGGUUUGUG | hmo-miR-15a | UAGCAGCACAGAAUGGUUUGUG |
| cca-miR-15b | UAGCAGCACAUCAUGGUUUGUA | hno-miR-15b | UAGCAGCACAUCAUGGUUUGUA | hmo-miR-15b | UAGCAGCACAUCAUGGUUUGUA |
|  |  | hno-miR-15c | AAGCAGCGCGUCAUGGUUUUC |  |  |
| cca-miR-16a | UAGCAGCACGUAAAUAUUGGUG | hno-miR-16a | UAGCAGCACGUAAAUAUUGGUG | hmo-miR-16a | UAGCAGCACGUAAAUAUUGGUG |
| cca-miR-16b | UAGCAGCACGUAAAUAUUGGAG | hno-miR-16b | UAGCAGCACGUAAAUAUUGGAG | hmo-miR-16b | UAGCAGCACGUAAAUAUUGGAG |
| cca-miR-16c | UAGCAGCAUGUAAAUAUUGGA |  |  | hmo-miR-16c | UAGCAGCAUGUAAAUAUUGGAG |
| cca-miR-17-5p | CAAAGUGCUUACAGUGCAGGUAG |  |  |  |  |
| cca-miR-181a-5p | AACAUUCAACGCUGUCGGUGA | hno-miR-181a | AACAUUCAACGCUGUCGGUGAGU | hmo-miR-181a | AACAUUCAACGCUGUCGGUGAGU |
| cca-miR-181b | AACAUUCAUUGCUGUCGGUGG | hno-miR-181b | AACAUUCAUUGCUGUCGGUGGG | hmo-miR-181b | AACAUUCAUUGCUGUCGGUGGG |
| cca-miR-181c | CACAUUCAUUGCUGUCGGUGGG | hno-miR-181c | CACAUUCAUUGCUGUCGGUGGG | hmo-miR-181c | CACAUUCAUUGCUGUCGGUGGG |
| cca-miR-182-5p | UUUGGCAAUGGUAGAACUCACAC | hno-miR-182 | UUUGGCAAUGGUAGAACUCACA | hmo-miR-182 | UUUGGCAAUGGUAGAACUCACA |
| cca-miR-183 | UAUGGCACUGGUAGAAUUCACUG | hno-miR-183 | UAUGGCACUGGUAGAAUUCACUG | hmo-miR-183 | UAUGGCACUGGUAGAAUUCACUG |
| cca-miR-184 | UGGACGGAGAACUGAUAAGGGC | hno-miR-184 | UGGACGGAGAACUGAUAAGGGC | hmo-miR-184 | UGGACGGAGAACUGAUAAGGGC |
| cca-miR-187 | UCGUGUCUUGUGUUGCAGCCAGU | hno-miR-187 | UCGUGUCUUGUGUUGCAGCC | hmo-miR-187 | UCGUGUCUUGUGUUGCAGCC |
| cca-miR-18a | UAAGGUGCAUCUAGUGCAGAUAG |  |  |  |  |
| cca-miR-18b | UAAGGUGCAUUUAGUGCAGAUAG |  |  |  |  |
| cca-miR-18c | UAAGGUGCAUCUUGUGUAGUUAG | hno-miR-18c | UAAGGUGCAUCUUGUGUAGUUA | hmo-miR-18c | UAAGGUGCAUCUUGUGUAGUUA |
| cca-miR-190 | UGAUAUGUUUGAUAUAUUAGG |  |  |  |  |
| cca-miR-192 | AUGACCUAUGAAUUGACAGCC | hno-miR-192 | AUGACCUAUGAAUUGACAGCC | hmo-miR-192 | AUGACCUAUGAAUUGACAGCC |
| cca-miR-193a | AACUGGCCUACAAAGUCCCAGU | hno-miR-193a | AACUGGCCUACAAAGUCCCAGU | hmo-miR-193a | AACUGGCCUACAAAGUCCCAGU |
| cca-miR-193b | AACUGGCCCGCAAAGUCCCGCU | hno-miR-193b | AACUGGCCCGCAAAGUCCCGCU | hmo-miR-193b | AACUGGCCCGCAAAGUCCCGCU |
| cca-miR-194 | UGUAACAGCAACUCCAUGUGGA | hno-miR-194a | UGUAACAGCAACUCCAUGUGG | hmo-miR-194a | UGUAACAGCAACUCCAUGUGG |
| cca-miR-196a | UAGGUAGUUUCAUGUUGUUGGG | hno-miR-196a | UAGGUAGUUUCAUGUUGUUGGG | hmo-miR-196a | UAGGUAGUUUCAUGUUGUUGGG |
| cca-miR-196b | UAGGUAGUUUCAAGUUGUUGGG | hno-miR-196b | UAGGUAGUUUCAAGUUGUUGGG | hmo-miR-196b | UAGGUAGUUUCAAGUUGUUGGG |
| cca-miR-199-5p | CCCAGUGUUCAGACUACCUGUUC | hno-miR-199 | CCCAGUGUUCAGACUACCUGUUC | hmo-miR-199 | CCCAGUGUUCAGACUACCUGUUC |
| cca-miR-19a | UGUGCAAAUCUAUGCAAAACUGA |  |  |  |  |
| cca-miR-19b-3p | UGUGCAAAUCCAUGCAAAACUGA | hno-miR-19b | UGUGCAAAUCCAUGCAAAACUGA | hmo-miR-19b | UGUGCAAAUCCAUGCAAAACUGA |
| cca-miR-19c | UGUGCAAAUCCAUGCAAAACUCG | hno-miR-19c | UGUGCAAAUCCAUGCAAAACUCG | hmo-miR-19c | UGUGCAAAUCCAUGCAAAACUCG |
| cca-miR-19d | UGUGCAAACCCAUGCAAAACUGA | hno-miR-19d | UGUGCAAACCCAUGCAAAACUGA | hmo-miR-19d | UGUGCAAACCCAUGCAAAACUGA |
| cca-miR-200a | UAACACUGUCUGGUAACGAUG | hno-miR-200a | UAACACUGUCUGGUAACGAUGU | hmo-miR-200a | UAACACUGUCUGGUAACGAUGU |
| cca-miR-200b | UAAUACUGCCUGGUAAUGAUGA | hno-miR-200b | UAAUACUGCCUGGUAAUGAUGA | hmo-miR-200b | UAAUACUGCCUGGUAAUGAUGA |
| cca-miR-200c | UAAUACUGCCUGGUAAUGAUGC | hno-miR-200c | UAAUACUGCCUGGUAAUGAUGC | hmo-miR-200c | UAAUACUGCCUGGUAAUGAUGC |
|  |  | hno-miR-202 | AGAGGCAUAGGGCAUGGGAAAA | hmo-miR-202 | AGAGGCAUAGGGCAUGGGAAAA |
| cca-miR-203a | GUGAAAUGUUUAGGACCACUUG | hno-miR-203a | GUGAAAUGUUUAGGACCACUUG | hmo-miR-203a | GUGAAAUGUUUAGGACCACUUG |
| cca-miR-203b-3p | GUGAAAUGUUCAGGACCACUUG | hno-miR-203b | GUGAAAUGUUCAGGACCACUUG | hmo-miR-203b | GUGAAAUGUUCAGGACCACUUG |
| cca-miR-204 | UUCCCUUUGUCAUCCUAUGCCU | hno-miR-204 | UUCCCUUUGUCAUCCUAUGCCU | hmo-miR-204 | UUCCCUUUGUCAUCCUAUGCCU |
| cca-miR-205 | UCCUUCAUUCCACCGGAGUCUG | hno-miR-205 | UCCUUCAUUCCACCGGAGUCUG | hmo-miR-205 | UCCUUCAUUCCACCGGAGUCUG |
| cca-miR-206 | UGGAAUGUAAGGAAGUGUGUGG | hno-miR-206 | UGGAAUGUAAGGAAGUGUGUGG | hmo-miR-206 | UGGAAUGUAAGGAAGUGUGUGG |
| cca-miR-20a-5p | UAAAGUGCUUAUAGUGCAGGUAG |  |  |  |  |
| cca-miR-20b | CAAAGUGCUCACAGUGCAGGUAG | hno-miR-20b | CAAAGUGCUCACAGUGCAGGUAG | hmo-miR-20b | CAAAGUGCUCACAGUGCAGGUAG |
| cca-miR-21 | UAGCUUAUCAGACUGGUGUUGGC | hno-miR-21 | UAGCUUAUCAGACUGGUGUUGGC | hmo-miR-21 | UAGCUUAUCAGACUGGUGUUGGC |
| cca-miR-210-3p | CUGUGCGUGUGACAGCGGCU | hno-miR-210 | CUGUGCGUGUGACAGCGGCUAA | hmo-miR-210 | CUGUGCGUGUGACAGCGGCUAA |
|  |  | hno-miR-212 | UAACAGUCUACAGUCAUGGCU | hmo-miR-212 | UAACAGUCUACAGUCAUGGCU |
| cca-miR-214 | UACAGCAGGCACAGACAGG | hno-miR-214 | ACAGCAGGCACAGACAGGCAG | hmo-miR-214 | ACAGCAGGCACAGACAGGCAG |
| cca-miR-216a | UAAUCUCAGCUGGCAACUGUGA | hno-miR-216a | UAAUCUCAGCUGGCAACUGUGA | hmo-miR-216a | UAAUCUCAGCUGGCAACUGUGA |
| cca-miR-216b | UAAUCUCUGCAGGCAACUGUGA | hno-miR-216b | UAAUCUCUGCAGGCAACUGUGA | hmo-miR-216b | UAAUCUCUGCAGGCAACUGUGA |
| cca-miR-217 | UACUGCAUCAGGAACUGAUUGGA | hno-miR-217 | UACUGCAUCAGGAACUGAUUGG | hmo-miR-217 | UACUGCAUCAGGAACUGAUUGG |
|  |  | hno-miR-2184 | AACAGUAAGAGUUUAUGUGCU | hmo-miR-2184 | AACAGUAAGAGUUUAUGUGCU |
|  |  | hno-miR-2187 | UUACAGGCUAUGCUAAUCUAUG |  |  |
|  |  | hno-miR-2188 | AAGGUCCAACCUCACAUGUCC | hmo-miR-2188 | AAGGUCCAACCUCACAUGUCC |
| cca-miR-218a | UUGUGCUUGAUCUAACCAUGUG | hno-miR-218a | UUGUGCUUGAUCUAACCAUGUG | hmo-miR-218a | UUGUGCUUGAUCUAACCAUGUG |
| cca-miR-218b | UUGUGCUUGAUCUAACCAUGC | hno-miR-218b | UUGUGCUUGAUCUAACCAUGCA | hmo-miR-218b | UUGUGCUUGAUCUAACCAUGCA |
|  |  | hno-miR-219 | UGAUUGUCCAAACGCAAUUCUU | hmo-miR-219 | UGAUUGUCCAAACGCAAUUCUU |
| cca-miR-221 | AGCUACAUUGUCUGCUGGG | hno-miR-221 | AGCUACAUUGUCUGCUGGGUUUC | hmo-miR-221 | AGCUACAUUGUCUGCUGGGUUUC |
| cca-miR-222 | AGCUACAUCUGGCUACUGGG | hno-miR-222 | AGCUACAUCUGGCUACUGGGUCUC | hmo-miR-222 | AGCUACAUCUGGCUACUGGGUCUC |
| cca-miR-223 | UGUCAGUUUGUCAAAUACCCCA | hno-miR-223 | UGUCAGUUUGUCAAAUACCCC | hmo-miR-223 | UGUCAGUUUGUCAAAUACCCC |
| cca-miR-22a | AAGCUGCCAGCUGAAGAACUGU | hno-miR-22a | AAGCUGCCAGCUGAAGAACUGU | hmo-miR-22a | AAGCUGCCAGCUGAAGAACUGU |
| cca-miR-22b | AAGCUGCCAGUUGAAGAGCUGU | hno-miR-22b | AAGCUGCCAGUUGAAGAGCUGU | hmo-miR-22b | AAGCUGCCAGUUGAAGAGCUGU |
| cca-miR-23a | AUCACAUUGCCAGGGAUUUCC | hno-miR-23a | AUCACAUUGCCAGGGAUUUCCA | hmo-miR-23a | AUCACAUUGCCAGGGAUUUCCA |
| cca-miR-23b | AUCACAUUGCCAGGGAUUACC | hno-miR-23b | AUCACAUUGCCAGGGAUUACCA | hmo-miR-23b | AUCACAUUGCCAGGGAUUACCA |
| cca-miR-24 | UGGCUCAGUUCAGCAGGAACAG | hno-miR-24 | UGGCUCAGUUCAGCAGGAACAG | hmo-miR-24 | UGGCUCAGUUCAGCAGGAACAG |
| cca-miR-25 | CAUUGCACUUGUCUCGGUCUGA | hno-miR-25 | CAUUGCACUUGUCUCGGUCUGA | hmo-miR-25 | CAUUGCACUUGUCUCGGUCUGA |
| cca-miR-26a | UUCAAGUAAUCCAGGAUAGGCU | hno-miR-26a | UUCAAGUAAUCCAGGAUAGGCU | hmo-miR-26a | UUCAAGUAAUCCAGGAUAGGCU |
| cca-miR-26b | UUCAAGUAAUCCAGGAUAGGUU | hno-miR-26b | UUCAAGUAAUCCAGGAUAGGUU | hmo-miR-26b | UUCAAGUAAUCCAGGAUAGGUU |
| cca-miR-27a | UUCACAGUGGCUAAGUUCCGC | hno-miR-27a | UUCACAGUGGCUAAGUUCCGCU | hmo-miR-27a | UUCACAGUGGCUAAGUUCCGCU |
| cca-miR-27b | UUCACAGUGGCUAAGUUCUGC | hno-miR-27b | UUCACAGUGGCUAAGUUCUGCA | hmo-miR-27b | UUCACAGUGGCUAAGUUCUGCA |
| cca-miR-27c-3p | UUCACAGUGGUUAAGUUCUGCC |  |  |  |  |
| cca-miR-27d | UUCACAGUGGCUAAGUUCUUC | hno-miR-27d | UUCACAGUGGCUAAGUUCUUCA | hmo-miR-27d | UUCACAGUGGCUAAGUUCUUCA |
| cca-miR-27e | UUCACAGUGGCUAAGUUCAGU | hno-miR-27e | UUCACAGUGGCUAAGUUCAGUG | hmo-miR-27e | UUCACAGUGGCUAAGUUCAGUG |
| cca-miR-29a | UAGCACCAUUUGAAAUCGGUUA | hno-miR-29a | UAGCACCAUUUGAAAUCGGUUA | hmo-miR-29a | UAGCACCAUUUGAAAUCGGUUA |
| cca-miR-29b | UAGCACCAUUUGAAAUCAGUGUU | hno-miR-29b | UAGCACCAUUUGAAAUCAGUGU | hmo-miR-29b | UAGCACCAUUUGAAAUCAGUGU |
| cca-miR-301a | CAGUGCAAUAGUAUUGUCAAAGC | hno-miR-301a | CAGUGCAAUAGUAUUGUCAAAG | hmo-miR-301a | CAGUGCAAUAGUAUUGUCAAAG |
| cca-miR-301b | CAGUGCAAUAGUAUUGUCAUUGC | hno-miR-301b | CAGUGCAAUAGUAUUGUCAUUG | hmo-miR-301b | CAGUGCAAUAGUAUUGUCAUUG |
| cca-miR-301c | CAGUGCAAUAGUAUUGUCAUAGC | hno-miR-301c | CAGUGCAAUAGUAUUGUCAUAG | hmo-miR-301c | CAGUGCAAUAGUAUUGUCAUAG |
| cca-miR-30a | UGUAAACAUUCCCGACUGGAAG |  |  |  |  |
| cca-miR-30b | UGUAAACAUCCUACACUCAGCU | hno-miR-30b | UGUAAACAUCCUACACUCAGCU | hmo-miR-30b | UGUAAACAUCCUACACUCAGCU |
| cca-miR-30c | UGUAAACAUCCUACACUCUCAGC | hno-miR-30c | UGUAAACAUCCUACACUCUCAG | hmo-miR-30c | UGUAAACAUCCUACACUCUCAG |
| cca-miR-30d | UGUAAACAUCCCCGACUGGAAGC | hno-miR-30d | UGUAAACAUCCCCGACUGGAAG | hmo-miR-30d | UGUAAACAUCCCCGACUGGAAG |
| cca-miR-30e-5p | UGUAAACAUCCUUGACUGGAAGC | hno-miR-30e | UGUAAACAUCCUUGACUGGAAG | hmo-miR-30e | UGUAAACAUCCUUGACUGGAAG |
| cca-miR-31 | UGGCAAGAUGUUGGCAUAGCUG |  |  |  |  |
| cca-miR-338 | UCCAGCAUCAGUGAUUUUGUUG | hno-miR-338 | UCCAGCAUCAGUGAUUUUGUUG | hmo-miR-338 | UCCAGCAUCAGUGAUUUUGUUG |
| cca-miR-34 | UGGCAGUGUCUUAGCUGGUUGU | hno-miR-34 | UGGCAGUGUCUUAGCUGGUUGU | hmo-miR-34 | UGGCAGUGUCUUAGCUGGUUGU |
|  |  | hno-miR-34b | UAGGCAGUGUUGUUAGCUGAUUG | hmo-miR-34b | UAGGCAGUGUUGUUAGCUGAUUG |
| cca-miR-363 | AAUUGCACGGUAUCCAUCUGUA | hno-miR-363 | AAUUGCACGGUAUCCAUCUGUA | hmo-miR-363 | AAUUGCACGGUAUCCAUCUGUA |
| cca-miR-365 | UAAUGCCCCUAAAAAUCCUUAU | hno-miR-365 | UAAUGCCCCUAAAAAUCCUUAU | hmo-miR-365 | UAAUGCCCCUAAAAAUCCUUAU |
| cca-miR-375 | UUUGUUCGUUCGGCUCGCGUUA | hno-miR-375 | UUUGUUCGUUCGGCUCGCGUUA | hmo-miR-375 | UUUGUUCGUUCGGCUCGCGUUA |
| cca-miR-429 | UAAUACUGUCUGGUAAUGCCGU | hno-miR-429 | UAAUACUGUCUGGUAAUGCCGU | hmo-miR-429 | UAAUACUGUCUGGUAAUGCCGU |
|  |  | hno-miR-429b | UAAUACUGCCUGGUAAUGCCAU | hmo-miR-429b | UAAUACUGCCUGGUAAUGCCAU |
| cca-miR-430 | UAAGUGCUAUUUGUUGGGGUAG | hno-miR-430a | UAAGUGCUAUUUGUUGGGGUAG | hmo-miR-430a | UAAGUGCUAUUUGUUGGGGUAG |
|  |  | hno-miR-430b | AAAGUGCUAUCAAGUUGGGGUAG | hmo-miR-430b | AAAGUGCUAUCAAGUUGGGGUAG |
| cca-miR-451 | AAACCGUUACCAUUACUGAGU | hno-miR-451 | AAACCGUUACCAUUACUGAGUU | hmo-miR-451 | AAACCGUUACCAUUACUGAGUU |
| cca-miR-454a | UAGUGCAAUAUUGCUAAUAGG | hno-miR-454a | UAGUGCAAUAUUGCUAAUAGGG | hmo-miR-454a | UAGUGCAAUAUUGCUAAUAGGG |
| cca-miR-454b | UAGUGCAAUAUUGCUUAUAGG | hno-miR-454b | UAGUGCAAUAUUGCUUAUAGGG | hmo-miR-454b | UAGUGCAAUAUUGCUUAUAGGG |
| cca-miR-455 | UAUGUGCCCUUGGACUACAUCG | hno-miR-455 | UAUGUGCCCUUGGACUACAUCG | hmo-miR-455 | UAUGUGCCCUUGGACUACAUCG |
|  |  | hno-miR-455b | GUAUGUGCCCUUGGACUACAUU | hmo-miR-455b | GUAUGUGCCCUUGGACUACAUU |
| cca-miR-456 | CAGGCUGGUUAGAUGGUUGUCA | hno-miR-456 | CAGGCUGGUUAGAUGGUUGUCA | hmo-miR-456 | CAGGCUGGUUAGAUGGUUGUCA |
| cca-miR-457a | AGCAGCACAUCAAUAUUGGC | hno-miR-457a | AAGCAGCACAUCAAUAUUGGCA | hmo-miR-457a | AAGCAGCACAUCAAUAUUGGCA |
| cca-miR-457b | AGCAGCACAUAAAUACUGGAG | hno-miR-457b | AAGCAGCACAUAAAUACUGGAG | hmo-miR-457b | AAGCAGCACAUAAAUACUGGAG |
| cca-miR-458 | AUAGCUCUUUGAAUGGUACUGC | hno-miR-458 | AUAGCUCUUUGAAUGGUACUGC | hmo-miR-458 | AUAGCUCUUUGAAUGGUACUGC |
| cca-miR-459-5p | AGUAACAAGGAUUCAUCCUGUU |  |  |  |  |
| cca-miR-460-5p | CCUGCAUUGUACACACUGUGCG | hno-miR-460 | CCUGCAUUGUACACACUGUGCG | hmo-miR-460 | CCUGCAUUGUACACACUGUGCG |
| cca-miR-462 | UAACGGAACCCAUAAUGCAGCUG | hno-miR-462 | UAACGGAACCCAUAAUGCAGCU | hmo-miR-462 | UAACGGAACCCAUAAUGCAGCU |
| cca-miR-489 | UGACAUCAUAUGUACGGCUGCU | hno-miR-489 | AGUGACAUCAUAUGUACGGCUGC | hmo-miR-489 | AGUGACAUCAUAUGUACGGCUGC |
| cca-miR-499 | UUAAGACUUGCAGUGAUGUUUA | hno-miR-499 | UUAAGACUUGCAGUGAUGUUUA | hmo-miR-499 | UUAAGACUUGCAGUGAUGUUUA |
| cca-miR-722 | UUUUUUGCAGAAACGUUUCAG | hno-miR-722 | UUUUUUGCAGAAACGUUUCAGAUU | hmo-miR-722 | UUUUUUGCAGAAACGUUUCAGAUU |
| cca-miR-724 | UUAAAGGGAAUUUGCGACUGUU | hno-miR-724 | UUAAAGGGAAUUUGCGACUGUU | hmo-miR-724 | UUAAAGGGAAUUUGCGACUGUU |
| cca-miR-725 | UUCAGUCAUUGUUUCUAGUAGU | hno-miR-725 | UUCAGUCAUUGUUUCUAGUAGU | hmo-miR-725 | UUCAGUCAUUGUUUCUAGUAGU |
| cca-miR-726 | UUCACUACUAGCAGAACUCGG |  |  |  |  |
| cca-miR-727-3p | GUUGAGGCGAGUUGAAGACUUA | hno-miR-727 | GUUGAGGCGAGUUGAAGACUUA | hmo-miR-727 | GUUGAGGCGAGUUGAAGACUUA |
| cca-miR-729 | CAUGGGUAUGAUACGACCUGGG | hno-miR-728 | AUACUAAGUACACUACGUUUUC | hmo-miR-728 | AUACUAAGUACACUACGUUUUC |
| cca-miR-730 | UCCUCAUUGUGCAUGCUGUGUG |  |  |  |  |
| cca-miR-731 | AAUGACACGUUUUCUCCCGGAUC | hno-miR-731 | AAUGACACGUUUUCUCCCGGAUCG | hmo-miR-731 | AAUGACACGUUUUCUCCCGGAUCG |
|  |  | hno-miR-733 | UGCGUUGGUUUAGCUCAGUGGUU | hmo-miR-733 | UGCGUUGGUUUAGCUCAGUGGUU |
| cca-miR-734 | UAAAUGCUGCAGAAUCGUACCG | hno-miR-734 | GUAAAUGCUGCAGAAUCGUACCG | hmo-miR-734 | GUAAAUGCUGCAGAAUCGUACCG |
|  |  | hno-miR-736 | GUAAGACGAACAAAAAGUUUU | hmo-miR-736 | GUAAGACGAACAAAAAGUUUU |
| cca-miR-738 | GCUACGGCCCGCGUCGGGA |  |  |  |  |
|  |  | hno-miR-739 | AGGCCGAAGUGGAGAAGGGU | hmo-miR-739 | AGGCCGAAGUGGAGAAGGGU |
| cca-miR-7a | UGGAAGACUAGUGAUUUUGUUGUU | hno-miR-7a | UGGAAGACUAGUGAUUUUGUUGU | hmo-miR-7a | UGGAAGACUAGUGAUUUUGUUGU |
| cca-miR-7b | UGGAAGACUUGUGAUUUUGUUGU | hno-miR-7b | UGGAAGACUUGUGAUUUUGUU | hmo-miR-7b | UGGAAGACUUGUGAUUUUGUU |
| cca-miR-9-5p | UCUUUGGUUAUCUAGCUGUAUG | hno-miR-9 | UCUUUGGUUAUCUAGCUGUAUGA | hmo-miR-9 | UCUUUGGUUAUCUAGCUGUAUGA |
| cca-miR-92a | UAUUGCACUUGUCCCGGCCUGU |  |  |  |  |
| cca-miR-92b | UAUUGCACUCGUCCCGGCCUCC | hno-miR-92b | UAUUGCACUCGUCCCGGCCUCC | hmo-miR-92b | UAUUGCACUCGUCCCGGCCUCC |
| cca-miR-93 | AAAAGUGCUGUUUGUGCAGGUAG | hno-miR-93 | AAAAGUGCUGUUUGUGCAGGUA | hmo-miR-93 | AAAAGUGCUGUUUGUGCAGGUA |
| cca-miR-96 | UUUGGCACUAGCACAUUUUUGCU |  |  | hmo-miR-96 | UUUGGCACUAGCACAUUUUUGCU |
| cca-miR-99 | AACCCGUAGAUCCGAUCUUGU | hno-miR-99 | AACCCGUAGAUCCGAUCUUGUG | hmo-miR-99 | AACCCGUAGAUCCGAUCUUGUG |
| cca-miR-10a-3p | CAAAUUCGUGUCUUGGGGAAU |  |  |  |  |
| cca-miR-126-5p | CAUUAUUACUUUUGGUACGCG |  |  |  |  |
| cca-miR-133a-5p | AGCUGGUAAAAUGGAACCAAA |  |  |  |  |
| cca-miR-133b-5p | UGGUCAAAUGGAACCAAGUCAG |  |  |  |  |
| cca-miR-140-3p | UACCACAGGGUAGAACCACGGA |  |  |  |  |
| cca-miR-142a-3p | GUAGUGUUUCCUACUUUAUGG |  |  |  |  |
| cca-miR-15a-3p | CAGGCCGUACUGUGCUGCGG |  |  |  |  |
| cca-miR-17-3p | ACUGCAGUGGAGGCACUUCUAGC |  |  |  |  |
| cca-miR-181a-3p | ACCAUCGACCGUUGAUUGUACC |  |  |  |  |
| cca-miR-182-3p | GUGGUUCUAGACUUGCCAACUA |  |  |  |  |
| cca-miR-199-3p | ACAGUAGUCUGCACAUUGGUU |  |  |  |  |
| cca-miR-19b-5p | AGUUUUGCUGGUUUGCAUUCAG |  |  |  |  |
| cca-miR-202-5p | UUCCUAUGCAUAUACCUCUUUGA |  |  |  |  |
| cca-miR-203b-5p | AGUGGUUCUCAACAGUUCAACAG |  |  |  |  |
| cca-miR-20a-3p | ACUGCAGUGUGAGCACUUGAAGU |  |  |  |  |
| cca-miR-210-5p | AGCCACUGACUAACGCACAUUG |  |  |  |  |
| cca-miR-27c-5p | CAGGACUUAACCCACUUGUGAAC |  |  |  |  |
| cca-miR-30e-3p | CUUUCAGUCGGAUGUUUGCAGC |  |  |  |  |
| cca-miR-459-3p | CAGGGAAUCUCUGUUACUGGG |  |  |  |  |
| cca-miR-460-3p | CACAGCGCAUACAAUGUGGAUG |  |  |  |  |
| cca-miR-727-5p | UCAGUCUUCAAUUCCUCCCAGC |  |  |  |  |
| cca-miR-9-3p | UAAAGCUAGAUAACCGAAAGUA |  |  |  |  |
