# Supplementary material for: Identification and Profiling of MicroRNAs from Skeletal Muscle of the Common Carp
Source: PLoS One. 2012 Jan 27;7(1):e30925. doi: 10.1371/journal.pone.0030925 (PMC3267759; doi:10.1371/journal.pone.0030925)
Supplement: Table S4 — Primer sequences for RT-PCR and qPCR assays. (DOC) [file pone.0030925.s007.doc]

**Table S4.** Primer sequences for RT-PCR and qPCR assays.

| **miRNA** | **Primer sequence** |
| --- | --- |
| cca-let-7a | GCCGTGAGGTAGTAGGTTGTAT |
| cca-miR-1 | CCGTGGAATGTAAAGAAGTATGT |
| cca-miR-10a-5p | CCTGTAGATCCGAATTTGTG |
| cca-miR-21 | CGTAGCTTATCAGACTGGTGTT |
| cca-miR-23a | ATCACATTGCCAGGGATTTC |
| cca-miR-24 | TGGCTCAGTTCAGCAGGA |
| cca-miR-26a | TTCAAGTAATCCAGGATAG |
| cca-miR-27a | TTCACAGTGGCTAAGTTCCG |
| cca-miR-133a-3p | TGGTCCCCTTCAACCAGCTG |
| cca-miR-143 | CCTGAGATGAAGCACTGTAGC |
| cca-miR-146a | GCTGAGAACTGAATTCCATAG |
| cca-miR-181a-3p | AACATTCAACGCTGTCGGT |
| cca-miR-206 | CGTGGAATGTAAGGAAGTGTG |
| cca-miR-214 | TACAGCAGGCACAGACAGG |
| cca-miR-222 | CGAGCTACATCTGGCTACTG |
| reverse-Ambion | gcgagcacagaattaatacgac |
| u6 forward | CGCTTCGGCAGCACATATAC |
| u6 reverse | TTCACGAATTTGCGTGTCA |
